# Second-Child Fertility Intentions among Urban Women in China: A Systematic Review and Meta-Analysis

**DOI:** 10.3390/ijerph20043744

**Published:** 2023-02-20

**Authors:** Yu Yang, Rongxin He, Ning Zhang, Liming Li

**Affiliations:** 1 School of Humanities and Social Science, Xi’an Jiaotong University, Xi’an 710049, China; 2 Vanke School of Public Health, Tsinghua University, Haidian District, Beijing 100084, China; 3 School of Public Policy and Administration, Xi’an Jiaotong University, Xi’an 710049, China

**Keywords:** fertility intention, second child, urban women, China, meta-analysis

## Abstract

With the adjustment of China’s fertility policy, the topic of women’s fertility has attracted much attention. In particular, urban women face a difficult choice between family and work. This study analyzed the prevalence and determinants of second-child fertility intention among urban women in China and aimed to provide evidence for improving fertility rate measures. A systematic review and meta-analysis were conducted using quantitative primary studies. We identified 16 cross-sectional studies that investigated a total of 24,979 urban women. The prevalence of second-child fertility intentions was 37%. A subgroup analysis revealed that the highest prevalence was observed between 2016 and 2017, and the lowest was observed in first-tier cities. Meta-analyses indicated that 18 factors were significantly associated with second-child fertility intentions, including demographic factors, fertility attitude, husbands, children, parents, or others. The findings of this study highlight the low second-child fertility intentions among urban women in China. Therefore, policymakers should consider various aspects, gradually optimizing fertility-supporting facilities, while encouraging fertility.

## 1. Introduction

According to the World Population Prospects 2022 report by the United Nations, between 1950 and 2021, the global total fertility rate decreased from 4.86 to 2.32. Furthermore, the report predicted that the total number of older people worldwide will be more than double that of children under 5 by 2050 [1]. The decrease in births will lead to a decline in labor stock, a rapid increase in labor costs, and tremendous pressure on social security systems, which could hinder future social and economic development [2]. The persistent decreases in fertility rates has become a global problem.

China is different from other countries because its low fertility is a result of policy restrictions that subsequently caused low endogenous fertility [3]. China has implemented a one-child policy since the 1970s, and only one or two children in a family has become the norm in Chinese society. Since the introduction of this policy, China’s fertility rate has been falling and remains below the replacement level [4]. In response to falling fertility rates, policymakers began to loosen birth restrictions. At the beginning of 2016, the Chinese government implemented birth encouragement and a universal two-child policy [5]. However, according to data from the seventh census in China, the total fertility rate of women was 1.3 in 2020, showing that the fertility rate increased in the short term [6]. However, the two-child policy failed to pull China out of the low-fertility dilemma for a long time, which prompted a greater amount of research on this subject.

Behavioral intentions refer to whether a person is ready to perform a certain behavior [7]. According to research, fertility intention is a useful indicator of fertility behavior [8]. Existing research suggests that many factors could affect people’s fertility intention, such as economic insecurity [9,10], the recession [11], family and workplace policies [12,13], gender egalitarianism [14], air pollution [15], fertility costs [16], conceptual factors [17,18], partners’ educational pairings [19], etc. Additionally, the attitude of the first child and parents towards the second child [20], son preference [4], and public expenditure [21] were related to the intention to give birth to a second child. Analyzing the reasons for not wanting another child can help us to better understand people’s fertility intentions [11].

Furthermore, studies have shown that women in non-agricultural occupations had lower fertility intentions than women in agriculture [22]. Among them, the increase in the number of children has a more significant negative impact on urban women [23], especially young urban women [24]. According to existing research, the reasons for women’s fertility intentions include work–family conflict [25], unequal division of labor within the family [26], the surrounding environment and people [27,28] and the wish to continue the family line [29]. This trend is more pronounced in East Asian countries [30]. Moreover, after becoming mothers, women will face the motherhood wage penalty [31,32], health penalty [33], career breaks [34], and childcare burdens [35], which may reduce women’s fertility enthusiasm. Therefore, this paper focuses on urban women as the main research object.

At present, there are many studies on the second-child fertility intention (SFI) of urban women, but their conclusions often differ. For instance, some scholars believe that well-educated women are more likely to want more children than poorly educated women [36,37]. However, some scholars believe that female education level has a significant negative impact on the intention to give birth to a second child [38]; Modena F. argues that the precariousness of women’s occupations greatly impedes their decision to have children [9]. However, Gatta A. believes that the perception of employment stability has only a limited impact [39].

However, for now, there is not a systematic review of research findings, neither in Chinese nor in English languages. Therefore, conducting a systematic review of the influencing factors of Chinese urban women’s intention to give birth to a second child is necessary. This systematic review and meta-analysis aimed to examine the prevalence of SFI (intentions for a second child) and identify the related factors among urban women after fully liberalizing the second-child policy.

## 2. Materials and Methods

This study followed the preferred Reporting Items for Systematic Reviews and Meta-Analysis (PRISMA) guidelines [40], and MOOSE Checklist for Meta-analyses of Observational Studies.

### 2.1. Data Sources and Searches

The literature search carried out in this study was a citation search conducted by one author, including four English-language databases (PubMed, EMBASE, Science Direct, Web of Science) and three Chinese databases (CNKI, WANFANG DATA, VIP). Since 2012, China has begun to gradually implement a second-child policy. Therefore, this paper limited the time of the literature search to January 2012–March 2022. The search strategy was based on a combination of “(fertility intention, or fertility desire, or intention to give birth) AND (women, or female, or childbearing age women) AND (China or Chinese)”. References to the retrieved studies were also checked and screened. The complete search strategy can be found online in Supplementary Table S1. We screened the literatures from March 2022 to May 2022.

### 2.2. Study Selection

Eligible published studies that reported on the prevalence and associated determinants of SFI among urban women in China were included. The eligibility criteria included (1) types of studies: original cross-sectional studies (those presenting non-original data, such as reviews, editorials, opinion papers, or letters to the editor, were excluded); (2) types of participants: Chinese urban women (women who live in cities); (3) types of outcome measures: the prevalence of fertility intent for a second child and related factors reported in the study.

The articles finally included in this paper were selected by two reviewers independently after evaluating the titles, abstracts, and full texts of all articles retrieved from the search. Moreover, any disagreements regarding article selection were resolved through group consultation.

### 2.3. Data Extraction and Quality Assessment

We extracted primary data from the included articles according to a standardized scale (the Cochrane Effective Practice and Organization of Care Review Group data collection checklist), including author, year, publication year, study site, sample size, number of cases, assessment tools, prevalence of SFI, and related factors. One author performed data extraction, which was independently reviewed by two other authors, and disagreements were resolved through group consultation. The inter-rater reliability for title screening was 98.64% between two authors and 96.99% for the abstract screening. The total assessment result can be found online in Supplementary Table S2.

Cross-sectional studies were independently assessed for bias by three authors using the modified Newcastle Ottawa Scale [41], which included: representativeness of the sample, sample size, non-respondents, ascertainment of the exposure, comparability of subjects in different outcome groups, assessment of the outcome, and statistical test. The scale has a total score of 7 points, and the quality of articles can be divided into three levels according to the score: 1–2 is the lowest, 3–5 is moderate, and 6–7 is the best. Specifically, a study is considered to be less representative if it did not test the reliability and validity of the measurement tools used (score = 0, otherwise = 1). A cross-sectional study with a sample size of less than 776 (the median of all samples from the cross-sectional studies) is considered a low-quality study (score = 0, otherwise = 1). An article is rejected if it has a score below 3 points. The disagreements between the three authors on the scoring of cross-sectional studies were partially resolved through group consultation.

### 2.4. Data Synthesis and Statistical Analysis

This paper analyzes the prevalence and influencing factors of urban women’s intention to give birth to a second child. According to the factors in the literature, we divided these factors into three categories: individual level, family level, and social level.

We compared the differences in SFI among urban women from different years and research sites by subgroup analyses. In the research time group, according to the promulgation time of China’s birth policy, we divided the years into 2016–2017 and 2018–2020. In the research site group, cities are divided into first-tier cities, new first-tier cities, and others, according to their level of development.

This study presents the relationship between factors in urban women and SFI with meta-analysis. Two conditions need to be met in the meta-analysis: the studies must explore the correlation between identical factors and SFI, while ensuring the use of consistent quantification methods for each of these factors, and at least three articles must be related to the factors. We judged the significance of pooled OR by Z-test via a meta-analysis. Heterogeneity was estimated using the Q statistic and evaluated using the I^2^ statistic [42]. If heterogeneity between studies was low (I^2^ <= 50%), a fixed-effects model was used; otherwise (I^2^ > 50%), a random-effects model was used. Publication bias was evaluated using Egger’s test. All statistical analyses were performed using StataV.13.0 and RevMan V.5.4. A two-tailed *p*-value of <0.05 was considered statistically significant. We used a descriptive method to explain the SFI correlation among urban women for factors that could not be quantitatively integrated and meta-analyzed.

## 3. Results

### 3.1. Literature Search

**Figure 1:**
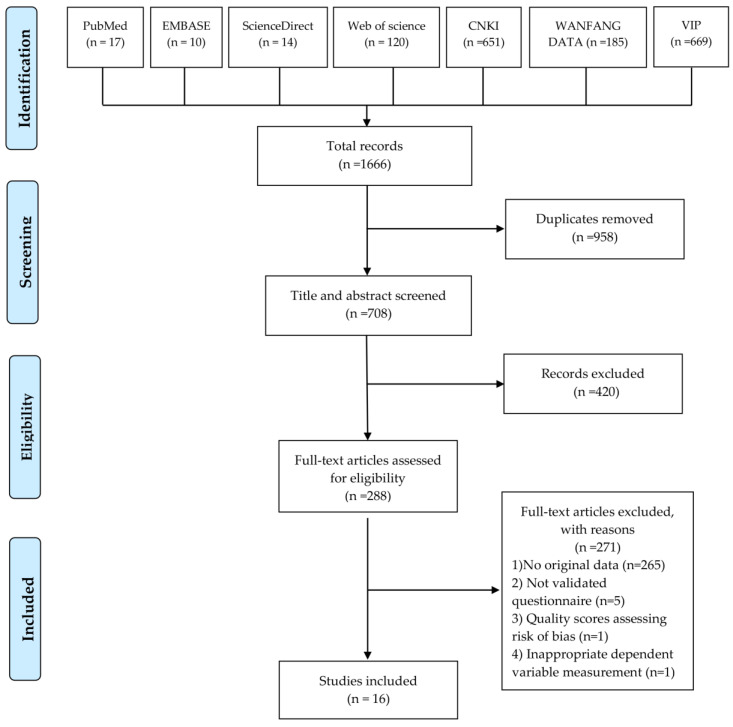
Flow diagram of study selection process.

We retrieved 1666 records from the initial database (PubMed: 17, Embase: 10, Science Direct: 14, Web of Science: 120, CNKI:651, WANFANG DATA:185, VIP: 669). After duplicate records were removed, 708 studies were screened by title and abstract, and 228 articles were used for full-text screening. Ultimately, a total of 16 articles were analyzed in this study. The types of excluded articles were divided into four categories: articles with a lack of original data (*n* = 265), articles that used unvalidated questionnaires (*n* = 5), articles that found scores of less than three points when assessing the risks of bias (*n* = 1), and articles with inappropriate measurements of the dependent variable (*n* = 1). In addition, our reference search revealed that the remaining 16 articles did not meet any of these exclusion criteria. The study selection process is shown in Figure 1.

### 3.2. Study Characteristics

The characteristics of the included studies are shown in Table 1. The selected studies were all cross-sectional and included 24,979 participants, with a median sample size of 776 (range: 213–12,722). All included surveys were conducted between 2016 and 2020. The research sites encompassed 24 cities in 12 provinces in China, spanning the eastern, central, and western regions of China. All studies used the question, “Would you like to give birth to a second child?”, to measure SFI, and the answers were divided into “Yes” and “No”.

This paper extracted a total of 65 factors from 16 studies (the results can be found online in the Supplementary Tables S3–S5). It included 3 groups: 22 personal environment factors (14 demographic factors; 7 fertility attitude factors), 38 family environment factors (18 husband factors; 11 child factors; 9 parents or others factors), and 5 social environmental factors (3 policy factors; 2 public service factors). The average quality score of the 16 included studies was four out of six points, indicating a moderate research quality according to the modified Newcastle–Ottawa Scale. All studies were of a medium and high quality. (Supplementary Table S6, available online)

### 3.3. Principal Findings

Table 2 shows the prevalence of SFI among urban women in China. The pooled prevalence was 37%. In these articles, the highest prevalence is 69% [50], while lowest prevalence is only 5% [51]. The subgroup analysis by research time showed that 2016–2017 (41%) has a higher prevalence than 2018–2020 (34%). Due to the differences between cities, this paper also conducts a subgroup analysis of city levels. The results show the highest prevalence in others (43%), followed by new first-tier cities (39%) and first-tier cities (29%). Due to differences in the study site and time, the included studies have a significant heterogeneity.

### 3.4. Meta-Analysis

#### 3.4.1. Factors Related to SFI among Urban Women

The 16 articles included in this study all reported attributable factors. Among a total of 65 factors, 18 factors were included in the meta-analysis. There were eight individual level factors and ten family level factors. Egger’s linear regression test on a natural logarithm scale of OR found no evidence of publication bias for the studies included in meta-analyses. The results can be found online in Supplementary Table S7.

#### 3.4.2. Individual Level Factors and SFI

The individual level of this study consists of two parts: demographic factors and fertility attitude (urban women’s attitude towards to give birth to a second child). Regarding demographic factors, the meta-analysis was based on 13 cross-sectional studies. According to the features of the included literature, all variables were treated as dichotomies.

**Figure 2:**
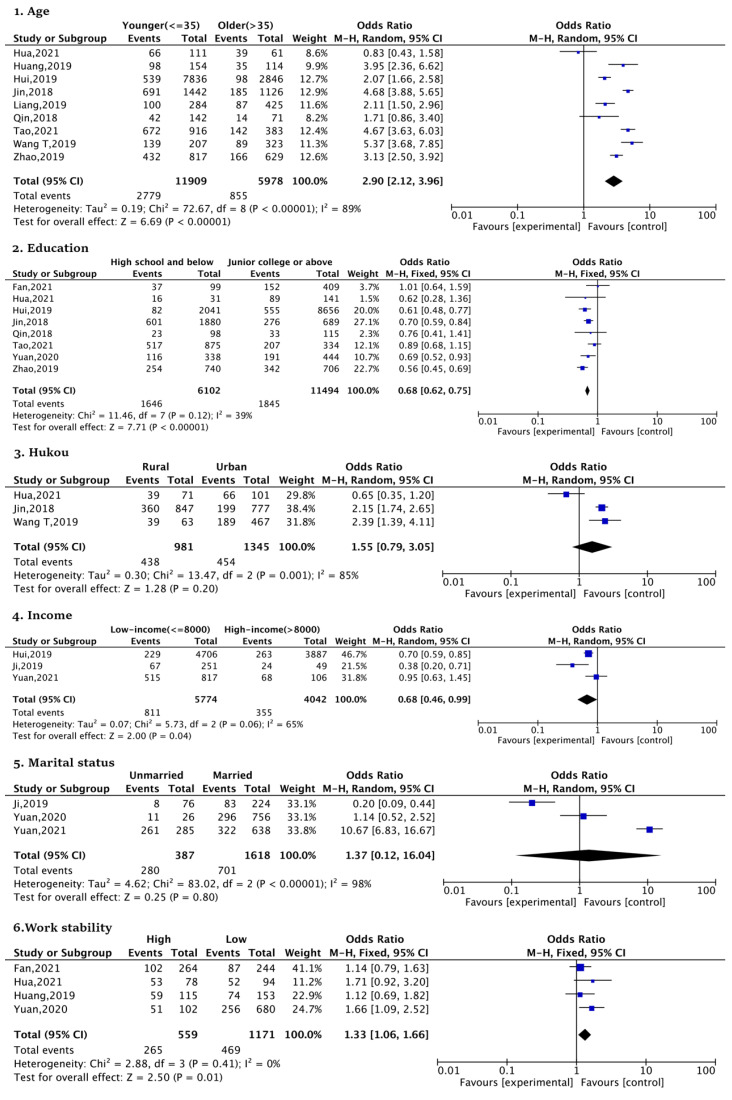
Forest plots of demographic factors [20,43,44,45,46,47,48,49,51,53,54,55,56].

Age (younger (<=35 years old) vs. older (>35 years old), OR:2.90; 95%CI), education (high school and below vs. junior college or above, OR:0.68), income (low-income (<=8000 yuan) vs. high-income (>8000 yuan), OR:0.68), and work stability (high vs. low, OR:1.33) were significantly associated with SFI in urban women. The results show that younger urban women who had a higher education, higher income, and stable job were more likely to give birth to a second child. However, hukou (rural vs. urban, OR:1.55) (government certificate of legal residency) [20] and marital status (unmarried vs. married, OR:1.37) were not statistically significant (Figure 2).

**Figure 3:**
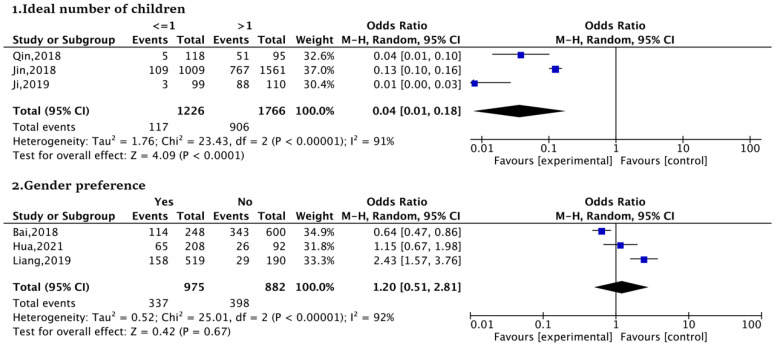
Forest plots of fertility attitude [44,47,53,54,55,57].

Regarding fertility attitude, the meta-analysis was based on six studies. The ideal number of children (<=1 vs. >1, OR:0.04) was significantly associated with SFI in urban women; when an urban women’s ideal number of children is greater than one, they are more likely to give birth to a second child. Gender preference (Yes vs. No, OR:1.20) was not statistically significant (Figure 3).

#### 3.4.3. Family Level Factors and SFI

Fourteen studies explored the association between family level and SFI. We divided the family level into three groups: husband factors, children factors, parents or others factors.

**Figure 4:**
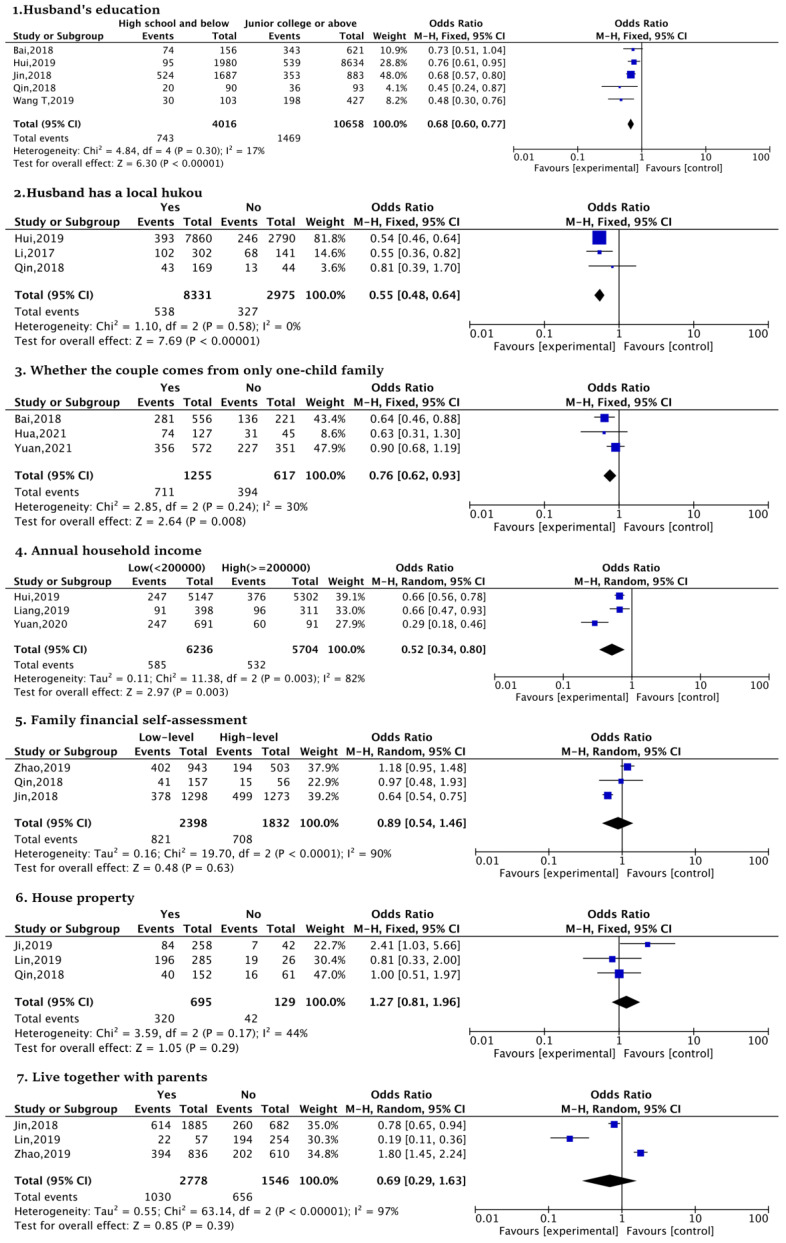
Forest plots of husband factors [20,43,44,46,47,49,50,51,52,53,54,55,57].

Firstly, husband factors, such as the husband’s education (high school and below vs. junior college, OR:0.68), the possession of a local hukou (yes vs. no, OR:0.55), whether the couple comes from only one-child family (yes vs. no, OR:0.76), and annual household income (low (<200,000 yuan) vs. high (≥200,000 yuan), OR:0.52) were significantly associated with SFI in urban women. This revealed that urban women with a higher prevalence of SFI are those who have a husband with a higher education, local hukou, and higher annual household income; moreover, the couple does not come from a one-child family. However, family financial self-assessment (low-level vs. high-level, OR:0.89), house property (yes vs. no, OR:1.27), and living together with parents (yes vs. no, OR:0.69) were not statistically significant factors (Figure 4).

**Figure 5:**
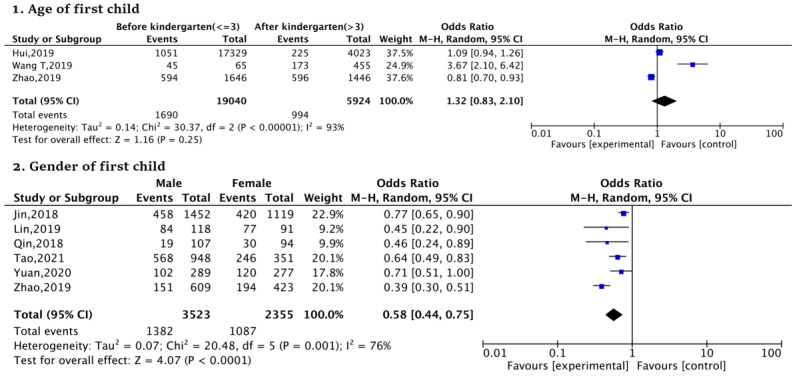
Forest plots of children factors [20,46,49,50,51,54,55,56].

Secondly, in children factors, the gender of the first child (male vs. female, OR:0.58) was significantly associated with SFI in urban women, which showed that urban women whose first child was female are more likely to give birth to a second child. Nevertheless, the age of the first child (before kindergarten (<=3 years old) vs. after kindergarten (>3 years old), OR:1.32) was not statistically significant (Figure 5).

**Figure 6:**
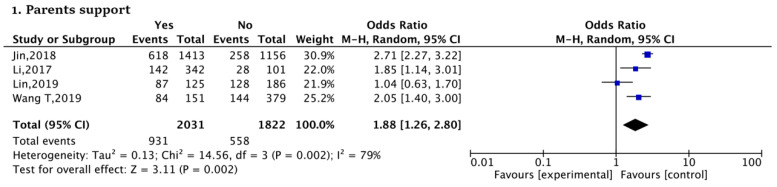
Forest plots of parents or others factors [20,50,52,55].

Finally, in parents or others factors, parents’ support (yes vs. no, OR:1.88) (Parents providing assistance with child rearing or financial help) was significantly associated with SFI in urban women. The results show that urban women who had parental support had a higher prevalence of SFI (Figure 6).

For the remaining 47 exposures, we illustrate their associations using the SFI of urban women under a narrative approach. These results can be found online in Supplementary Table S8.

On an individual level, in demographic factors, urban women who come from a one-child family [50], physical health [45], frequency of participation in community activities [49], and work pressure [52] demonstrated significant differences. However, those in management positions [44], job title [46], nationality [48], and employment status [55] demonstrate no significant differences. Regarding fertility attitude, urban women of ideal childbearing age [50], age at birth of first child [46], impact of childbirth on women’s careers [45], and facing conflicting choices between career and family [44,47] demonstrated significant differences. However, the expected sex of the first child and the expected sex of the two children [44] demonstrated no significant differences.

On a family level, regarding husband factors, husband’s age [20], hukou [52], income [54], fertility attitude [20], and household registration type of the couple [56] had significant differences. The factors regarding whether the husband is from a one-child family [54,55], husband ‘s occupation [51,54], job stability [51], the number of desired children of the spouse or family [44,55], and marital satisfaction [50] had no significant differences. Regarding children factors, the health condition of the first child [50], method of childcare [44,50], cost of raising children or childcare [44,47,51], husband’s time spent on housework and child care [49], and fertility attitudes of the first child [20] had significant differences. However, babysitting fees [51] had no significant differences. Regarding parents or others factors, the fertility attitude of parents, in-laws and friends [20], and envy of other people’s second child [50] had significant differences. However, giving birth to a second child due to parents’ influences, such as birth pressure [50], number of children [55], gender of children [55], and in-law relationships [52], had no significant differences.

On a social level, the attitude of women’s companies (the attitudes of companies towards women having a second child) [44,47], women’s awareness of the universal two-child policy (how much women know about the two-child policy) [44,47], women’s fertility intention before the implementation of the universal two-child policy, public medical and pension insurance [44], and the state of supervision of childcare institutions [47] had significant differences.

## 4. Discussion

This systematic review of 16 cross-sectional studies and 24,979 participants showed an overall prevalence of SFI (37%) among urban women. The results of subgroup analysis show that, since the implementation of the two-child policy, SFI first increased and then decreased. From 2016 to 2017, SFI showed an upward trend. However, over time, the influence of policy has declined. The analysis results also show that the degree of urban development is inversely proportional to the fertility intention of urban women; the more developed the city, the lower the fertility intention. In the meta-analysis, we found that urban women’s age, education, income, job stability, and ideal number of children can significantly affect their fertility intention. In addition, husband’s education, possession of a local hukou, whether the couple comes from a one-child family, annual household income, sex of first child, and parental support also affect urban women’s intention to give birth to a second child.

In terms of the fertility intention of urban women in China, 37% of women have SFI, which is close to that of some developed countries. A fertility study of Austrian women showed that 39% of all young respondents wanted two children [58]. A fertility study conducted in Poland showed that almost 40% of women had two or more children [59]. A Canadian study including 11,001 participants showed that the average intention to give birth to a second child was 48% [60]. Among East Asian countries, the intention of Korean working women with one child to give birth to a second child is 38.39%, and results are similar in China [13].

The results of the subgroup analysis showed differences in two aspects. In research time, since the two-child policy was implemented in China, the intention to give birth to a second child in 2016–2017 was significantly higher than that in 2018–2020. This result shows that the new policy promoted fertility in 2016–2017. However, with the extension of time, there is a marginal diminishing effect. According to China’s National Bureau of Statistics, from 2016 to 2021, the country’s birth rate showed a trend of first increasing and then decreasing [61]. This is consistent with the research trend of this paper. Due to China’s long-term one-child policy, many couples have been forced to suppress their desire to have children. However, with the implementation of the second-child policy, this pent-up desired was released, causing a high number of births in the few years after the policy was implemented, before returning to the previous birth rate, and thus forming stacking effect of fertility [62]. This was also confirmed by a cross-sectional study from China, which showed that relaxing the family planning policies does increase the residents’ intention to have more children in the short term [63].

In terms of research sites, women in first-tier cities have the lowest intention to give birth to a second child, followed by new first-tier cities, while other cities have the highest SFI. Specifically, the more economically developed the city, the lower the intention of women to have children. The result is consistent with the actual situation in China, where women in first-tier cities experience a faster pace of life, a more competitive labor market, and more expensive housing [64]. This dampens the enthusiasm of urban women to give birth to a second child. This geographic feature of fertility also exists in Australia and Korea. There is a distinct geographic pattern whereby the total fertility rate is about 0.5 higher in remote and very remote areas in Australia (2.33) compared to major cities (1.82) [65]. In South Korea, fertility rates have also sharply decreased in some major cities, such as Seoul or Pusan [66].

On an individual level, we conclude that younger women with a higher income, higher education, more stable jobs, and more than one ideal number of children had a significantly higher prevalence of SFI, which is consistent with previous research conclusions. Research suggests that the ideal childbearing age is between 28 and 35 years [67]. Older women face a higher risk than younger women of giving birth to babies. In addition, there is a strong relationship between education, income, and job stability. Traditional research claims that the improvement of women’s education level is inversely proportional to their fertility level [68]. The findings of this study are consistent with Testa, Maria Rita (2014), who described a positive association between women’s level of education and lifetime fertility intentions [37]. Furthermore, the increased risk of unemployment can inhibit women’s intention to have children [69]. A steady job means a stable income. In China, women working in government departments, state-owned enterprises, and public institutions are more willing to give birth to a second child since the low risk of unemployment increases the sense of security for women to give birth. Especially in recent years, the impact of the COVID-19 pandemic on the global economy has increased the risk of unemployment in the labor market of private sector. Therefore, the advantage of job security in the public sector is especially important.

On a family level, urban women whose husbands have a higher education, higher annual household income, and not possession a local hukou—and where the couple is not from a one-child family, the first child is a girl, and parental support is available—had a significantly higher prevalence of SFI. Specifically, couples with a college degree or above are more likely to give birth to a second child. This result shows that educational pairings influence women’s intentions to have additional children. When highly educated women have a highly educated partner, their fertility intentions are higher than lower-educated women [19]. At the same time, we also found that families with higher family incomes showed more enthusiasm for giving birth to a second child. This is justified by the fact that, under the prevailing circumstances of high living costs, long working hours, and burgeoning childcare expenses, raising a child in China has become far more expensive [70].

Based on this research, we made four additional interesting findings. Firstly, women have a higher intention to give birth to a second child if their husbands do not have a local hukou. Secondly, couples who are not from one-child families are more likely to give birth to a second child, which verifies the conclusion that the intergenerational transmission of fertility and that couples from extended families are more likely to have extended families of their own [71,72]. Thirdly, women whose first child is a girl are more likely to give birth to a second child. In urban areas of China, there is a tendency for couples who have a male child as their firstborn to exhibit a decreased willingness to have a second child [73]. Due to the traditional concept of marriage, the cost of raising boys is much higher than that of girls, which reduces people’s preference for having male children to a certain extent. From this conclusion, the Chinese preference for male children has changed, but not completely gone away. Finally, since the conflict between family and work always exists, and the support of parents for their children’s family can help women alleviate this conflict, there is no doubt that the family support of parents for their children will promote women’s fertility. In fact, grandparents’ participation in the care of young grandchildren in China is considerably high compared to many other societies [74].

In general, factors such as age, income, job stability, education, and the ideal number of children are important factors that affect the intention of women around the world to bear children. In addition, the fertility policy also plays a role, but whether it will continue to have an impact is questionable.

## 5. Conclusions

In conclusion, Chinese urban women’s intention to give birth to a second child is low. Their fertility intention is influenced by individuals, families, and society. On the one hand, the result reflects that urban women in China exhibit greater rationality when considering the option of having a second child. Fewer and better births are becoming the consensus of the new generation of childbearing age groups. On the other hand, it also reveals that structural problems in Chinese society have virtually undermined women’s desire to give birth to more children. Therefore, it is not wise for policy makers to only encourage people to have a second child.

We believe that the Government should pay more attention to the construction of social environments. For example, at the level of public construction, policy makers should establish and improve childcare services, optimize the education system, and improve the social pension mechanism. At the level of system construction, decision makers should improve the maternity leave system and combat gender discrimination in enterprises. At a cultural level, policy makers should promote gender equality, family responsibility sharing, etc. To create a childbearing-friendly social environment, it is possible to eliminate urban women’s reproductive concerns and work–family conflicts. In addition, due to the unbalanced ratio of men to women, we also recommend that the Government pays more attention to poor marriage practices in China. There is less tolerance of unmarried couples having children in East Asian cultures; therefore, marriage has become necessary for young people to have children.

## 6. Limitations, Strengths and Literature Gaps

This article has two advantages. First, it is the first systematic review of SFI among urban Chinese women. It is based on 16 cross-sectional studies covering a large sample of 24,979 participants, providing evidence from mainland China for a worldwide study of fertility intentions. Second, it explains the coldness of China’s two-child policy. Due to China’s long-standing one-child policy, policymakers have not had the expected results after the policy began to have a comprehensive two-child policy. Fertility is a complex issue, and there are more factors behind it than simple policy guidance. This paper explores the broad association of potential factors.

Our research also has limitations. One limitation was that some high heterogeneity was found when performing subgroup analyses and meta-analyses due to differences in study time and location. The other limitation is that, due to the limited number of included articles, this paper is unable to make a more detailed classification of urban women; for example, married women with one child, married women without children, and unmarried women. Due to the limited number of literatures included in this paper, we are unable to further explore the specific meaning of fertility intention, which is also a pity of this paper.

From the conclusions of this paper, we can see that there are still many possibilities for further research on fertility intention. First, this paper only focused on one definition of reproductive decision making, further research could focus on papers which explore the predictors of different constructs such as short vs. long-term intentions, fertility desires or ideal number of children, etc. Second, China has a large area and uneven economic development, so there are specific differences in the intention of urban women to bear children between regions. Third, in addition to the intuitive economic, number, and other factors of family factors, the gender relationship within the family and the reproductive pressure from parents are also aspects that need to be studied. Fourth, from a cultural perspective, we need an explanation of how the collision between China’s traditional fertility culture and modern fertility culture will shape the concept of the fertility among young people. Finally, the long-lasting COVID-19 pandemic may change people’s way of living and implicitly change people’s concept of fertility. This is a global problem that cannot be ignored.

## Figures and Tables

**Table 1:** Characteristics of 16 included studies.

Authors	Location	Number (Qualified Rate %)	Prevalence of FI, N Persons (%)	Ref. No.
Yuan, 2021 [43]	Xi’an	1152 (96.00%)	583 (50.6%)	213
Hua, 2021 [44]	Nanjing	305 (95.80%)	133 (43.61%)	186
Fan, 2021 [45]	Zhengzhou	508 (84.70%)	189 (37.2%)	177
Yuan, 2020 [46]	Ningbo	972 (97.20%)	307 (31.60%)	144
Ji, 2019 [47]	Qingdao	300 (93.75%)	91 (30.30%)	137
Huang, 2019 [48]	Changchun	268 (94.04%)	133 (49.63%)	134
Zhao, 2019 [49]	Beijing	1446 (95.40%)	598 (41.22%)	132
Lin, 2019 [50]	Putian	311 (94.24%)	215 (69.13%)	128
Hui, 2019 [51]	Shanghai	12,722 (99.76%)	641 (5.00%)	121
Li, 2017 [52]	Chengdu	523 (96.00%)	170 (32.50%)	109
Liang, 2019 [53]	Shanghai	904 (98.30%)	187 (20.70%)	95
Qin, 2018 [54]	Nanchong	213 (100.00%)	56 (26.30%)	87
Jin, 2018 [55]	12 cities of 6 provinces *	2799 (100.00%)	876 (31.3%)	83
Tao, 2021 [56]	Guilin	1708 (94.89%)	814 (47.66%)	62
Bai, 2018 [57]	Shanghai	848 (80.76%)	417 (49.17%)	21
Wang T, 2019 [20]	Hunan province	703 (82.40%)	228 (32.43%)	4

* Twelve cities (Guangzhou, Jieyang, Chengdu, Luzhou, Wuhan, Jingzhou, Jinan, Jining, Hangzhou, Lishui, Shenyang, Chaoyang). Six provinces (Guangdong, Sichuan, Hubei, Shandong, Zhejiang, Liaoning).

**Table 2:** Prevalence of SFI among urban women in China.

Variables	Characteristic	Include Studies	Prevalence (95%CI)	Q Test (I2) (%)
Overall		16	0.374 (0.259 to 0.488)	99.7
By research time	2016–2017	8	0.406 (0.322 to 0.490)	98.3
	2018–2020	8	0.340 (0.159 to 0.522)	99.8
By research site	First-tier cities *	4	0.290 (0.065 to 0.515)	99.8
	New first-tier cities **	5	0.389 (0.306 to 0.473)	95.0
	Others	6	0.427 (0.321 to 0.534)	97.9

* First-tier cities: traditional ranking of Chinese cities, the first-tier cities include Beijing, Shanghai, Guangzhou, and Shenzhen. ** New first-tier cities: The rankings are based on CBN weekly in 2022; the new first-tier cities include Chengdu, Hangzhou, Chongqing, Wuhan, Xi’an, Suzhou, Tianjin, Nanjing, Changsha, Zhengzhou, Dongguan, Qingdao, Shenyang, Ningbo, Kunming.

## Data Availability

Not applicable.

## Supplementary Materials

The following supporting information can be downloaded at: https://www.mdpi.com/article/10.3390/ijerph20043744/s1. Table S1: Search strategy; Table S2: The inter-rater reliability for the title abstract screening by the two authors; Table S3: 65 factors related to SFI. Table S4: Distribution of factors in 16 included studies. Table S5: Data extraction according to each factor. Table S6: Quality scores assessing risk of bias using a modified Newcastle-Ottawa scale; Table S7: Meta-analyses on 18 factors. Table S8: Factors not included in the meta-analysis and whether they were significantly associated with SFI.
