# Supplementary material for: Second-Child Fertility Intentions among Urban Women in China: A Systematic Review and Meta-Analysis
Source: Int J Environ Res Public Health. 2023 Feb 20;20(4):3744. doi: 10.3390/ijerph20043744 (PMC9962327; doi:10.3390/ijerph20043744)
Supplement: Supplementary file 1 [file ijerph-20-03744-s001.zip › ijerph-2094431-supplementary.pdf]

**Table S1 Search strategy**

**Database 1: PubMed**

| Sequence | Query |
|---|---|
| #1 | Search: **(China[Title/Abstract]) OR (Chinese[Title/Abstract])** |
| #2 | Search: **((fertility desire[Title/Abstract]) OR (fertility intention[Title/Abstract])) OR (willingness to give-birth[Title/Abstract])** |
| #3 | Search: **((women[Title/Abstract]) OR (female[Title/Abstract])) OR (childbearing age women[Title/Abstract])** |
| #4 | Search: **("2012/01/01"[Date - Publication] : "2022/03/09"[Date - Publication])** |
| #5 | #1AND#2 AND#3 AND#4 AND#5<br>Search: **(("2012/01/01"[Date - Publication] : "2022/03/09"[Date - Publication])) AND ((((china[Title/Abstract]) OR (Chinese[Title/Abstract])) AND (((fertility desire[Title/Abstract]) OR (fertility intention[Title/Abstract])) OR (willingness to give-birth[Title/Abstract]))) AND (((women[Title/Abstract]) OR (female[Title/Abstract])) OR (childbearing age women[Title/Abstract])))** |

**Database 2: EMBASE**

| Sequence | Query |
|---|---|
| #1 | **China:ab,ti OR Chinese:ab,ti** |
| #2 | **'fertility desire':ab,ti OR 'fertility intention':ab,ti OR 'willingness to give birth':ab,ti** |
| #3 | **women:ab,ti OR female:ab,ti OR 'childbearing age women':ab,ti** |

| | |
|---|---|
| **#4** | #1 AND #2 AND #3 |

**Database 3: ScienceDirect**

| **Sequence** | **Query** |
|---|---|
| **#1** | Find articles with these terms：<br>(fertility intention or fertility desire or willingness to give birth)and(women or female or childbearing age women)and(China or Chinese) |
| **#2** | Year：2012-2022 |
| **#3** | Article type：review articles and research articles |
| **#4** | #1 AND #2 AND #3 |

**Database 4: Web of science**

| **Sequence** | **Query** |
|---|---|
| **#1** | TS=(china) OR TS=(Chinese ) |
| **#2** | TS=(fertility desire ) OR TS=(fertility intention) OR TS=(willingness to give-birth) |
| **#3** | TS=(women) OR TS=(childbearing age women) OR TS=(female) |
| **#4** | Publication Date: All years(1898-2022) |
| **#5** | #1 AND #2 AND #3 AND #4<br>（TS=(china) OR TS=(Chinese )）AND（TS=(fertility desire ) OR TS=(fertility intention) OR TS=(willingness to reproduce)）AND（TS=(women) OR TS=(childbearing age women)） |

**Database 5: CAJD (CNKI)**
Time range: 2012-01-01---2022-03-09
TKA=('再生育意愿'+'生育意愿'+'生育意向'+'生育计划') AND TKA=('二胎'+'二孩'+'三胎'+'三孩') AND TKA=('妇女'+'女性'+'女')
**(English) Database 5: CAJD (CNKI)**
Time range: January 1, 2012--- March 9, 2022
TKA=('the intention to fertility again' + 'fertility intention'+' fertility desire'+' fertility plan') AND TKA=('Second child'+' Third child') AND TKA=('women '+'female')

**Database 6: CSPD (WANFANG Data)**

((题名或关键词: (二胎) or 题名或关键词: (二孩) or 题名或关键词: (三胎) or 题名或关键词: (三孩) )and (题名或关键词:(再生育意愿) or 题名或关键词:(生育意愿) or 题名或关键词:(生育意向) or 题名或关键词:(生育计划))and (题名或关键词:(女性) or 题名或关键词:(妇女) or 题名或关键词:(女))) and Date:2012-*

**(English) Database 6: CSPD (WANFANG Data)**

((title or key word: (Second child) or title or key word: (Third child))and (title or key word:( the intention to fertility again) or title or key word:(fertility intention) or title or key word:( fertility desire) or title or key word:( fertility plan))and (title or key word:(female) or title or key word:( women))) and Date:2012-*
**Database 7: VIP**
**Time range：2012-2022**
(U=(生育意愿 OR 生育意向 OR 生育计划 OR 再生育意向 )) AND ( U=(女性 OR 妇女 OR 女 )) AND ( U=(二胎 OR 二孩 OR 三胎 OR 三孩 ))
**(English) Database 7: VIP**
**Time range：2012-2022**
(U=( fertility intention OR fertility desire OR fertility plan OR the intention to fertility again )) AND ( U=( female OR women)) AND ( U=( Second child OR Third child))
Since Database 5-7 is the Chinese database included in the literature search in this paper, the search terms listed in it are in Chinese, so there is no need to translate them into English.

**Table S2 The inter-rater reliability for the title abstract screening by the two authors**

| | Title | | Abstract | |
|---|---|---|---|---|
| | Author 1 | Author 2 | Author 1 | Author 2 |
| Inclued | 475 | 428 | 243 | 264 |
| Exclued | 227 | 236 | 187 | 201 |
| Agreement | 655 | | 451 | |
| Sum | 664 | | 465 | |
| Rate | 98.64% | | 96.99% | |

**Table S3 65 factors related to SFI**

| Group | Category | Exposure |
|---|---|---|
| A **Individual level** | **Demographic factors** | A1-Age, A2-Education, A3-Hukou, A4-Income, A5-Marrige status, A6- Work stability,A7- Hold a management position,A8-Job title,A9- Nationality,A10-Whether comes from only one child family,A11- Physical health, A12-Frequency of participation in community activities,A13- Employed,A14- Work pressure . |
| | **Fertility attitude** | A15- Ideal childbearing age,A16- Ideal number of children,A17- Gender preference,A18- Age at the first child birthed,A19- Expected sex of first child,A20- Expected sex of two children, A21- The impact of childbirth on women's careers,A22- Facing conflicting choices between career and fertility. |
| B **Family level** | **Husband factors** | B1-Husband's age, B2- Husband's education, B3- Husband's hukou,B4- The husband has a local hukou,B5- Husband's occupation,B6- Husband's job stability,B7- Husband's income,B8-Whether husband from only one child family ,B9-Fertility attitude of husband,B10- Husband's desired number of children.B11- Household registration type of the couple,B12- Whether the couple comes from only one-child family,B13- Annual household income,B14- Family financial self-assessment,B15- House property,B16- Live together with parents, B17- Who influences fertility intention,B18-Marital satisfaction. |
| | **Children factors** | B19-Number of children being raised ,B20- Sex of children being raised,B21- Age of first child,B22- Gender of first child,B23-The health condition of first child, B24-The way of children care,B25-The cost of raising children, B26-Babysitting fees,B27-Childcare costs, B28-Husband’s time spent on housework and child care,B29-Fertility attitudes of the first child. |
| | **Parents or others factors** | B30-Parents support ,B31-To have second child pressure from parents,B32-Parent’s fertility attitude,B33-Parents’ desired number of children,B34-Whether the first child’s gender matches the parents’ expectations,B35-In-laws’ fertility attitude,B36- In-law relationships. B37-Envy of other people's second child, B38-Friend's fertility attitude. |

| | | |
|---|---|---|
| C **Social level** | **Policy factors** | C1-The attitude of women’s companies, C2-Women's awareness of the universal two-child policy, C3-Women's fertility intention before the implementation of the universal two-child policy. |
| | **Public service** | C4- The perfection of medical and pension insurance in public places, C5- The state of supervision of child care institutions. |

**Table S4 Distribution of factors in 16 included studies (1 = Yes, 0 = No)**

| Exposure | 16 included studies (Ref. No.) | | | | | | | | | | | | | | | | Total |
|---|---|---|---|---|---|---|---|---|---|---|---|---|---|---|---|---|---|
| | 4 | 21 | 62 | 83 | 87 | 95 | 109 | 121 | 128 | 132 | 134 | 137 | 144 | 177 | 186 | 213 | |
| **A1** | 1 | 0 | 1 | 1 | 1 | 1 | 0 | 1 | 0 | 1 | 1 | 1 | 1 | 0 | 1 | 1 | **12** |
| **A2** | 0 | 0 | 1 | 1 | 1 | 1 | 0 | 1 | 1 | 1 | 1 | 1 | 1 | 1 | 1 | 0 | **12** |
| **A3** | 1 | 0 | 0 | 1 | 0 | 0 | 0 | 0 | 0 | 0 | 0 | 0 | 0 | 0 | 1 | 0 | **3** |
| **A4** | 0 | 0 | 0 | 0 | 0 | 0 | 0 | 1 | 0 | 0 | 0 | 1 | 0 | 1 | 0 | 1 | **4** |
| **A5** | 0 | 0 | 0 | 0 | 0 | 0 | 0 | 0 | 0 | 0 | 0 | 1 | 1 | 0 | 0 | 1 | **3** |
| **A6** | 0 | 0 | 0 | 0 | 1 | 0 | 0 | 1 | 1 | 0 | 1 | 1 | 1 | 1 | 1 | 0 | **8** |
| **A7** | 0 | 0 | 0 | 0 | 0 | 0 | 0 | 0 | 0 | 0 | 0 | 0 | 0 | 0 | 1 | 0 | **1** |
| **A8** | 0 | 0 | 0 | 0 | 0 | 0 | 0 | 0 | 0 | 0 | 0 | 0 | 1 | 0 | 0 | 0 | **1** |
| **A9** | 0 | 0 | 0 | 0 | 0 | 0 | 0 | 0 | 0 | 0 | 1 | 0 | 0 | 0 | 0 | 0 | **1** |
| **A10** | 0 | 0 | 0 | 1 | 0 | 0 | 0 | 0 | 1 | 0 | 0 | 0 | 0 | 0 | 0 | 0 | **2** |
| **A11** | 0 | 0 | 0 | 0 | 0 | 0 | 0 | 0 | 1 | 0 | 0 | 0 | 0 | 1 | 0 | 0 | **2** |
| **A12** | 0 | 0 | 0 | 0 | 0 | 0 | 0 | 0 | 0 | 1 | 0 | 0 | 0 | 0 | 0 | 0 | **1** |
| **A13** | 0 | 0 | 0 | 1 | 0 | 0 | 0 | 0 | 0 | 0 | 0 | 0 | 0 | 0 | 0 | 0 | **1** |
| **A14** | 0 | 0 | 0 | 0 | 0 | 0 | 1 | 0 | 0 | 0 | 0 | 0 | 0 | 0 | 0 | 0 | **1** |
| **A15** | 0 | 0 | 0 | 0 | 0 | 0 | 0 | 0 | 1 | 0 | 0 | 0 | 0 | 0 | 0 | 0 | **1** |
| **A16** | 0 | 0 | 0 | 1 | 1 | 0 | 0 | 0 | 0 | 0 | 0 | 1 | 0 | 0 | 0 | 1 | **4** |
| **A17** | 0 | 1 | 0 | 0 | 0 | 1 | 0 | 0 | 1 | 0 | 0 | 0 | 0 | 0 | 1 | 0 | **4** |
| **A18** | 0 | 0 | 0 | 0 | 0 | 0 | 0 | 0 | 0 | 0 | 0 | 0 | 1 | 0 | 0 | 0 | **1** |
| **A19** | 0 | 0 | 0 | 0 | 0 | 0 | 0 | 0 | 0 | 0 | 0 | 0 | 0 | 0 | 1 | 0 | **1** |
| **A20** | 0 | 0 | 0 | 0 | 0 | 0 | 0 | 0 | 0 | 0 | 0 | 0 | 0 | 0 | 1 | 0 | **1** |
| **A21** | 0 | 0 | 0 | 0 | 0 | 0 | 0 | 0 | 0 | 0 | 0 | 0 | 0 | 1 | 1 | 0 | **2** |
| **A22** | 0 | 0 | 0 | 0 | 0 | 0 | 0 | 0 | 0 | 0 | 0 | 1 | 0 | 0 | 1 | 0 | **2** |
| **B1** | 1 | 0 | 0 | 0 | 1 | 0 | 0 | 0 | 0 | 0 | 0 | 0 | 0 | 0 | 0 | 0 | **2** |
| **B2** | 1 | 1 | 0 | 1 | 1 | 0 | 0 | 1 | 0 | 0 | 0 | 0 | 0 | 0 | 0 | 0 | **5** |
| **B3** | 0 | 0 | 0 | 0 | 0 | 0 | 1 | 0 | 0 | 0 | 0 | 0 | 0 | 0 | 0 | 0 | **1** |
| **B4** | 0 | 0 | 0 | 0 | 1 | 0 | 1 | 1 | 0 | 0 | 0 | 0 | 0 | 0 | 0 | 0 | **3** |
| **B5** | 0 | 0 | 0 | 0 | 1 | 0 | 0 | 1 | 0 | 0 | 0 | 0 | 0 | 0 | 0 | 0 | **2** |
| **B6** | 0 | 0 | 0 | 0 | 0 | 0 | 0 | 1 | 0 | 0 | 0 | 0 | 0 | 0 | 0 | 0 | **1** |
| **B7** | 0 | 0 | 0 | 0 | 1 | 0 | 0 | 0 | 0 | 0 | 0 | 0 | 0 | 0 | 0 | 0 | **1** |
| **B8** | 0 | 0 | 0 | 1 | 1 | 0 | 0 | 0 | 0 | 0 | 0 | 0 | 0 | 0 | 0 | 0 | **2** |

| | | | | | | | | | | | | | | | | |
|---|---|---|---|---|---|---|---|---|---|---|---|---|---|---|---|---|
| **B9** | 1 | 0 | 0 | 0 | 0 | 0 | 0 | 0 | 0 | 0 | 0 | 0 | 0 | 0 | 0 | 0 | **1** |
| **B10** | 0 | 0 | 0 | 1 | 0 | 0 | 0 | 0 | 0 | 0 | 0 | 0 | 0 | 0 | 0 | 0 | **1** |
| **B11** | 0 | 0 | 1 | 0 | 0 | 0 | 0 | 0 | 0 | 0 | 0 | 0 | 0 | 0 | 0 | 0 | **1** |
| **B12** | 1 | 1 | 0 | 0 | 0 | 1 | 0 | 0 | 0 | 0 | 0 | 0 | 0 | 0 | 1 | 1 | **5** |
| **B13** | 0 | 0 | 1 | 0 | 0 | 1 | 0 | 1 | 1 | 0 | 1 | 0 | 1 | 0 | 1 | 0 | **7** |
| **B14** | 0 | 0 | 0 | 1 | 1 | 0 | 0 | 0 | 0 | 1 | 0 | 0 | 0 | 0 | 0 | 0 | **3** |
| **B15** | 0 | 0 | 0 | 0 | 1 | 0 | 0 | 0 | 1 | 0 | 0 | 1 | 0 | 0 | 0 | 0 | **3** |
| **B16** | 0 | 0 | 0 | 1 | 0 | 0 | 0 | 0 | 1 | 1 | 0 | 0 | 0 | 0 | 0 | 0 | **3** |
| **B17** | 0 | 0 | 0 | 0 | 0 | 0 | 0 | 0 | 0 | 0 | 0 | 0 | 0 | 0 | 1 | 0 | **1** |
| **B18** | 0 | 0 | 0 | 0 | 0 | 0 | 0 | 0 | 1 | 0 | 0 | 0 | 0 | 0 | 0 | 0 | **1** |
| **B19** | 0 | 1 | 0 | 0 | 0 | 0 | 0 | 1 | 1 | 0 | 0 | 1 | 1 | 0 | 1 | 1 | **7** |
| **B20** | 0 | 0 | 0 | 0 | 0 | 0 | 0 | 1 | 0 | 0 | 1 | 0 | 1 | 0 | 0 | 0 | **3** |
| **B21** | 1 | 0 | 0 | 0 | 0 | 0 | 0 | 1 | 0 | 1 | 0 | 0 | 0 | 0 | 0 | 0 | **3** |
| **B22** | 0 | 0 | 1 | 1 | 1 | 0 | 0 | 0 | 1 | 1 | 0 | 0 | 1 | 0 | 0 | 0 | **6** |
| **B23** | 0 | 0 | 0 | 0 | 0 | 0 | 0 | 0 | 1 | 0 | 0 | 0 | 0 | 0 | 0 | 0 | **1** |
| **B24** | 0 | 0 | 0 | 0 | 0 | 0 | 0 | 0 | 1 | 0 | 0 | 0 | 0 | 0 | 1 | 0 | **2** |
| **B25** | 0 | 0 | 0 | 0 | 0 | 0 | 0 | 0 | 0 | 0 | 0 | 1 | 0 | 0 | 1 | 0 | **2** |
| **B26** | 0 | 0 | 0 | 0 | 0 | 0 | 0 | 1 | 0 | 0 | 0 | 0 | 0 | 0 | 0 | 0 | **1** |
| **B27** | 0 | 0 | 0 | 0 | 0 | 0 | 0 | 1 | 0 | 0 | 0 | 0 | 0 | 0 | 0 | 0 | **1** |
| **B28** | 0 | 0 | 0 | 0 | 0 | 0 | 0 | 0 | 0 | 1 | 0 | 0 | 0 | 0 | 0 | 0 | **1** |
| **B29** | 1 | 0 | 0 | 0 | 0 | 0 | 0 | 0 | 0 | 0 | 0 | 0 | 0 | 0 | 0 | 0 | **1** |
| **B30** | 1 | 0 | 0 | 1 | 0 | 0 | 1 | 0 | 1 | 0 | 0 | 0 | 0 | 0 | 0 | 0 | **4** |
| **B31** | 0 | 0 | 0 | 0 | 0 | 0 | 0 | 0 | 1 | 0 | 0 | 0 | 0 | 0 | 0 | 0 | **1** |
| **B32** | 1 | 0 | 0 | 0 | 0 | 0 | 0 | 0 | 0 | 0 | 0 | 0 | 0 | 0 | 0 | 0 | **1** |
| **B33** | 0 | 0 | 0 | 1 | 0 | 0 | 0 | 0 | 0 | 0 | 0 | 0 | 0 | 0 | 0 | 0 | **1** |
| **B34** | 0 | 0 | 0 | 1 | 0 | 0 | 0 | 0 | 0 | 0 | 0 | 0 | 0 | 0 | 0 | 0 | **1** |
| **B35** | 1 | 0 | 0 | 0 | 0 | 0 | 0 | 0 | 0 | 0 | 0 | 0 | 0 | 0 | 0 | 0 | **1** |
| **B36** | 0 | 0 | 0 | 0 | 0 | 0 | 1 | 0 | 0 | 0 | 0 | 0 | 0 | 0 | 0 | 0 | **1** |
| **B37** | 0 | 0 | 0 | 0 | 0 | 0 | 0 | 0 | 1 | 0 | 0 | 0 | 0 | 0 | 0 | 0 | **1** |
| **B38** | 1 | 0 | 0 | 0 | 0 | 0 | 0 | 0 | 0 | 0 | 0 | 0 | 0 | 0 | 0 | 0 | **1** |
| **C1** | 0 | 0 | 0 | 0 | 0 | 0 | 0 | 0 | 0 | 0 | 0 | 1 | 0 | 0 | 1 | 0 | **2** |
| **C2** | 0 | 0 | 0 | 0 | 0 | 0 | 0 | 0 | 0 | 0 | 0 | 1 | 0 | 0 | 1 | 0 | **2** |
| **C3** | 0 | 0 | 0 | 0 | 0 | 0 | 0 | 0 | 0 | 0 | 0 | 0 | 0 | 0 | 1 | 0 | **1** |

| | | | | | | | | | | | | | | | | | |
|---|---|---|---|---|---|---|---|---|---|---|---|---|---|---|---|---|---|
| **C4** | 0 | 0 | 0 | 0 | 0 | 0 | 0 | 0 | 0 | 0 | 0 | 0 | 0 | 0 | 1 | 0 | **1** |
| **C5** | 0 | 0 | 0 | 0 | 0 | 0 | 0 | 0 | 0 | 0 | 0 | 1 | 0 | 0 | 0 | 0 | **1** |
| **Total** | **12** | **4** | **5** | **15** | **13** | **5** | **5** | **14** | **17** | **8** | **6** | **13** | **10** | **5** | **20** | **6** | **158** |

**Table S5 Data extraction according to each factor**

***SFI: Second-child Fertility Intention; Non-SFI: Non Second-child Fertility Intention***

***A1-Age***

| Author | Group | SFI | Non-SFI | Results |
|---|---|---|---|---|
| **Yuan,2021** | <=25 | 78 | 46 | Significant difference ($\chi^2$=21.594, P<0.01). |
| | 26-30 | 187 | 117 | |
| | 31-35 | 189 | 107 | |
| | >=36 | 128 | 70 | |
| **Hua,2021** | 20-25 | 10 | 6 | No significant difference ($\chi^2$test). |
| | 25-30 | 39 | 24 | |
| | 30-35 | 17 | 15 | |
| | 35-40 | 17 | 9 | |
| | 40-45 | 10 | 8 | |
| | >=45 | 12 | 5 | |
| **Yuan,2020** | 20-29 | 119 | 201 | Significant difference ($\chi^2$=14.408, P<0.01). |
| | 30-39 | 179 | 250 | |
| | 40-48 | 9 | 24 | |
| **Ji,2019** | 20-25 | 3 | 44 | Significant difference ($\chi^2$=19.760, P<0.01). |
| | 26-30 | 38 | 81 | |
| | 31-35 | 28 | 35 | |
| | 36-40 | 13 | 23 | |
| | >40 | 9 | 26 | |
| **Huang,2019** | 20-30 | 79 | 27 | Significant difference ($\chi^2$=6.770, P<0.01). |
| | 31-40 | 38 | 58 | |
| | 40-50 | 16 | 50 | |
| **Zhao,2019** | 22-29 | 278 | 134 | No significant difference ($\chi^2$ test). |
| | 30-39 | 308 | 502 | |
| | 40-44 | 12 | 212 | |
| **Hui,2019** | <=24 | 22 | 164 | Significant difference ($\chi^2$=71.293, P<0.001). |
| | 25-29 | 232 | 2599 | |
| | 30-34 | 285 | 4534 | |
| | 35-39 | 89 | 2279 | |
| | >=40 | 9 | 469 | |
| **Liang,2019** | 25-29 | 27 | 37 | Significant difference ($\chi^2$=20.620, P<0.01). |
| | 30-34 | 73 | 147 | |
| | 35-39 | 54 | 178 | |
| | 40-46 | 33 | 160 | |
| **Qin,2018** | 20-29 | 31 | 58 | No significant difference ($\chi^2$ test). |
| | 30-39 | 22 | 85 | |
| | 40-49 | 3 | 14 | |

| | | | | |
|---|---|---|---|---|
| **Tao,2021** | <25 | 12 | 8 | Significant difference (P<0.05). |
| | 25-30 | 578 | 181 | |
| | 31-40 | 165 | 111 | |
| | >40 | 59 | 185 | |
| **Wang T,2019** | <31 | 79 | 23 | Significant difference ($\chi^2$=101.49 , P<0.001). |
| | 31-35 | 60 | 45 | |
| | >35 | 89 | 234 | |
| **Jin,2018** | 20-24 | 111 | 86 | No |
| | 25-29 | 326 | 279 | |
| | 30-34 | 254 | 386 | |
| | 35-39 | 132 | 473 | |
| | 40-44 | 53 | 468 | |

### *A2-Education*

| Author | Group | SFI | Non-SFI | Results |
|---|---|---|---|---|
| **Hua,2021** | Primary school and below | 1 | 0 | No significant difference ($\chi^2$test). |
| | Junior high school | 5 | 3 | |
| | High school | 10 | 12 | |
| | College degree | 32 | 21 | |
| | Undergraduate degree | 52 | 25 | |
| | Master degree and above | 5 | 6 | |
| **Fan,2021** | Junior high school and below | 12 | 21 | No significant difference ($\chi^2$test). |
| | high school or secondary school | 25 | 41 | |
| | College degree | 46 | 70 | |
| | Undergraduate degree | 80 | 138 | |
| | Master degree and above | 26 | 49 | |
| **Yuan,2020** | Junior high school and below | 62 | 73 | Significant difference ($\chi^2$=36.856, P<0.001). |
| | high school or secondary school | 54 | 149 | |
| | College degree or Higher vocational education | 69 | 136 | |
| | Undergraduate degree | 108 | 106 | |
| | Master degree or Doctor's Degree | 14 | 11 | |
| **Ji,2019** | Junior high school and below | 5 | 7 | No significant difference ($\chi^2$ test). |
| | High school or technical secondary school or vocational high school or technical school | 19 | 34 | |
| | College degree | 28 | 67 | |
| | Undergraduate degree | 33 | 81 | |
| | Master degree and above | 6 | 20 | |

| | | | | |
|---|---|---|---|---|
| **Huang,2019** | primary school | 7 | 3 | Significant difference ($\chi^2$=3.004, P<0.01). |
| | junior high school | 10 | 8 | |
| | High school or technical secondary school or technical school | 53 | 35 | |
| | College degree and above | 63 | 89 | |
| **Zhao,2019** | High school and below | 254 | 486 | Significant difference ($\chi^2$=10.71, P<0.01). |
| | College and above | 342 | 364 | |
| **Lin,2019** | Elementary school and below | 10 | 1 | No significant difference ($\chi^2$ test). |
| | junior high school | 50 | 20 | |
| | high school or secondary school | 46 | 20 | |
| | College degree or Undergraduate degree | 103 | 49 | |
| | Master degree and above | 6 | 6 | |
| **Hui,2019** | Elementary school and below | 4 | 55 | Significant difference ($\chi^2$=40.519, P<0.001). |
| | junior high school | 28 | 616 | |
| | high school or secondary school | 50 | 1288 | |
| | College degree | 173 | 2814 | |
| | Undergraduate degree | 296 | 4514 | |
| | Master degree and above | 86 | 773 | |
| **Liang,2019** | High school or technical secondary school and below | 17 | 36 | No significant difference ($\chi^2$ test). |
| | College degree | 35 | 124 | |
| | Undergraduate degree | 113 | 323 | |
| | Master degree and above | 22 | 39 | |
| **Qin,2018** | Junior high school and below | 13 | 33 | Significant difference ($\chi^2$=7.512, P<0.05). |
| | high school or college degree | 19 | 84 | |
| | Undergraduate degree and above | 24 | 40 | |
| **Tao,2021** | Junior high school and below | 301 | 250 | Significant difference (P<0.05). |
| | high school or secondary school or College degree | 325 | 163 | |
| | Undergraduate degree and above | 188 | 72 | |
| **Jin,2018** | Junior high school and below | 261 | 652 | No |
| | high school | 340 | 627 | |
| | College degree | 181 | 291 | |
| | Undergraduate degree and above | 95 | 122 | |

*A3-Hukou*

| Author | Group | SFI | Non-SFI | Results |
|---|---|---|---|---|
| **Hua,2021** | Non-rural Rural | 66<br>39 | 35<br>32 | No significant difference ($\chi^2$test). |

| | | | | |
|---|---|---|---|---|
| **Wang T,2019** | Rural | 39 | 24 | Significant difference ($\chi^2$=17.59, P<0.001). |
| | Non-rural | 189 | 278 | |
| **Jin,2018** | Rural | 360 | 487 | No |
| | Urban | 199 | 578 | |
| | resident | 317 | 627 | |

## *A4-Income*

| Author | Group | SFI | Non-SFI | Results |
|---|---|---|---|---|
| **Yuan,2021** | <=2000 | 112 | 80 | No significant difference ($\chi^2$test). |
| | 2001-4000 | 189 | 102 | |
| | 4001-6000 | 136 | 71 | |
| | 6001-8000 | 78 | 49 | |
| | >=8001 | 68 | 38 | |
| **Fan,2021** | <=2000 | 25 | 38 | No significant difference ($\chi^2$test). |
| | 2001-4000 | 12 | 21 | |
| | 4001-6000 | 25 | 41 | |
| | 6001-8000 | 46 | 70 | |
| | 8000-10000 | 80 | 138 | |
| | >=10000 | 26 | 49 | |
| **Ji,2019** | <3000 | 8 | 61 | Significant difference ($\chi^2$=14.989, P<0.01). |
| | 3000-5999 | 43 | 105 | |
| | 6000-9999 | 25 | 27 | |
| | 10000-20000 | 11 | 11 | |
| | >20000 | 4 | 5 | |
| **Hui,2019** | <4000 | 63 | 1219 | Significant difference ($\chi^2$=24.320, P<0.001). |
| | 4000-8000 | 166 | 3258 | |
| | 8001-12500 | 138 | 2233 | |
| | >12500 | 125 | 1391 | |

## *A5-Marriage status*

| Author | Group | SFI | Non-SFI | Results |
|---|---|---|---|---|
| **Yuan,2021** | Unmarried | 261 | 24 | Significant difference ($\chi^2$=171.249, P<0.001). |
| | Married | 322 | 316 | |
| **Yuan,2020** | Unmarried | 11 | 15 | No significant difference ($\chi^2$ test). |
| | Married | 296 | 460 | |
| **Ji,2019** | Unmarried | 8 | 68 | Significant difference ($\chi^2$=19.194, P<0.001). |
| | First marriage Remarried | 80 | 135 | |
| | divorced or widowed | 2 | 3 | |
| | | 1 | 3 | |

## *A6- Work stability*

| Author | Group | SFI | Non-SFI | Results |
|---|---|---|---|---|

| | | | | |
|---|---|---|---|---|
| **Hua,2021** | Government Department or Public Institution | 23 | 7 | Significant difference ($\chi^2$=24.855, P<0.01). |
| | State-owned enterprise or Collective enterprises | 30 | 18 | |
| | private enterprise | 45 | 22 | |
| | Freelancers | 7 | 20 | |
| **Fan,2021** | Government Department | 9 | 14 | No significant difference ($\chi^2$test). |
| | Public Institution | 87 | 123 | |
| | State-owned enterprise | 6 | 25 | |
| | Private enterprise | 47 | 86 | |
| | Others | 40 | 71 | |
| **Yuan,2020** | Government Department | 6 | 6 | No significant difference ($\chi^2$test). |
| | Public Institution | 45 | 45 | |
| | Enterprise | 104 | 164 | |
| | Individually-owned business | 66 | 107 | |
| | Freelancers | 43 | 77 | |
| | Unemployed | 9 | 28 | |
| | Others | 34 | 48 | |
| **Ji,2019** | Current students | 0 | 9 | Significant difference ($\chi^2$=20.824, P<0.01). |
| | State-owned enterprise | 7 | 28 | |
| | Private Enterprise | 26 | 29 | |
| | Foreign company or joint venture | 1 | 9 | |
| | Government Department or Public Institution | 20 | 74 | |
| | Individually-owned business | 7 | 19 | |
| | Freelancers | 9 | 15 | |
| | Housewife | 12 | 4 | |
| | Others | 9 | 22 | |
| **Huang,2019** | Government Department Public Institution | 59 | 56 | No significant difference ($\chi^2$ test). |
| | Enterprise | 40 | 40 | |
| | Service industry | 34 | 39 | |
| **Lin,2019** | Government Department | 47 | 29 | No significant difference ($\chi^2$ test). |
| | Freelancers | 68 | 36 | |
| | Private Enterprise | 46 | 18 | |
| | Housewife | 54 | 13 | |
| **Qin,2018** | Unemployed | 15 | 60 | No significant difference ($\chi^2$ test). |
| | Employed | 33 | 74 | |
| | Self-employed | 8 | 23 | |
| **Hui,2019** | Housewife | 142 | 1835 | Significant difference ($\chi^2$=6.633, P<0.05). |
| | Others | 497 | 8274 | |

*A7-Hold a management position*

| Author | Group | SFI | Non-SFI | Results |
|---|---|---|---|---|
| **Hua,2021** | Top management | 4 | 6 | No significant difference ($\chi^2$test). |
| | Middle managers | 11 | 5 | |
| | Grassroots managers | 20 | 17 | |
| | General staff | 70 | 39 | |

*A8-Job title*

| Author | Group | SFI | Non-SFI | Results |
|---|---|---|---|---|
| **Yuan,2020** | Senior title | 0 | 5 | No significant difference ($\chi^2$test). |
| | Deputy senior title | 4 | 5 | |
| | Intermediate title | 41 | 61 | |
| | Division title | 53 | 67 | |
| | No title | 209 | 337 | |

*A9-Nationality*

| Author | Group | SFI | Non-SFI | Results |
|---|---|---|---|---|
| **Huang,2019** | Han nationality | 123 | 129 | No significant difference ($\chi^2$test). |
| | Others | 10 | 6 | |

*A10-Whether comes from only one child family*

| Author | Group | SFI | Non-SFI | Results |
|---|---|---|---|---|
| **Jin,2018** | Yes | 516 | 1154 | No |
| | No | 361 | 540 | |
| **Lin,2019** | Yes | 17 | 19 | Significant difference ($\chi^2$=9.158, |
| | No | 198 | 77 | $P<0.01$). |

*A11- Physical health*

| Author | Group | SFI | Non-SFI | Results |
|---|---|---|---|---|
| **Fan,2021** | Very good | 59 | 64 | Significant difference ($\chi^2$=10.851, |
| | Good | 94 | 164 | $P<0.05$). |
| | Not too bad | 30 | 73 | |
| | Bad | 6 | 17 | |
| | Very bad | 0 | 1 | |
| **Lin,2019** | Very good | 182 | 71 | No significant difference ($\chi^2$ test). |
| | Good | 29 | 24 | |
| | Bad | 3 | 2 | |
| | Very bad | 1 | 0 | |

*A12- Frequency of participation in community activities*

| Author | Group | SFI | Non-SFI | Results |
|---|---|---|---|---|

| Author | Group | SFI | Non-SFI | Results |
|---|---|---|---|---|
| **Zhao,2019** | Once a month or none | 233 | 261 | Significant difference ($\chi^2$=22.78, P<0.001). |
| | At least twice a month | 363 | 589 | |

*A13- Employed*

| Author | Group | SFI | Non-SFI | Results |
|---|---|---|---|---|
| **Jin,2018** | Yes | 753 | 1538 | No |
| | No | 125 | 156 | |

*A14- Work pressure*

| Author | Group | SFI | Non-SFI | Results |
|---|---|---|---|---|
| **Li,2017** | Very high | 11 | 36 | Significant difference ($\chi^2$=-3.177[a], P<0.01). |
| | High | 43 | 94 | |
| | Not too bad | 70 | 102 | |
| | Low | 16 | 15 | |
| | Very low | 30 | 32 | |

*A15- Ideal childbearing age*

| Author | Group | SFI | Non-SFI | Results |
|---|---|---|---|---|
| **Lin,2019** | <20 | 0 | 1 | Significant difference ($\chi^2$=12.584, P<0.01). |
| | 20-25 | 86 | 38 | |
| | 26-30 | 128 | 51 | |
| | >30 | 1 | 6 | |

*A16-Ideal number of children*

| Author | Group | SFI | Non-SFI | Results |
|---|---|---|---|---|
| **Yuan,2021** | 0 | 0 | 11 | Significant difference ($\chi^2$=617.158, P<0.001). |
| | 1 | 152 | 327 | |
| | 2 | 429 | 2 | |
| | 3 | 2 | 0 | |
| **Ji,2019** | 0 | 0 | 8 | Significant difference ($\chi^2$=54.909, P<0.001). |
| | 1 | 3 | 88 | |
| | >=2 | 88 | 22 | |
| **Qin,2018** | 1 | 5 | 113 | Significant difference ($\chi^2$=66.401, P<0.001). |
| | >=2 | 51 | 44 | |
| **Jin,2018** | <=1 | 109 | 900 | No |
| | >=2 | 767 | 794 | |

*A17- Gender preference*

| Author | Group | SFI | Non-SFI | Results |
|---|---|---|---|---|
| **Hua,2021** | A girl | 2 | 29 | Significant difference ($\chi^2$=27.058, P<0.001). |
| | A boy | 1 | 23 | |
| | Boy and Girl | 55 | 87 | |
| | Two girls | 6 | 3 | |

| | | | | |
|---|---|---|---|---|
| | Two boys | 1 | 1 | |
| | No idea | 26 | 66 | |
| **Lin,2019** | A girl | 5 | 8 | Significant difference ($\chi^2$=26.977, P<0.001). |
| | A boy | 4 | 11 | |
| | Boy and Girl | 191 | 69 | |
| | Two girls | 1 | 4 | |
| | Two boys | 7 | 2 | |
| | Others | 7 | 2 | |
| **Liang,2019** | A girl | 14 | 49 | Significant difference ($\chi^2$=51.842, P<0.001). |
| | A boy | 26 | 120 | |
| | Boy and Girl | 118 | 192 | |
| | No idea | 29 | 161 | |
| **Bai,2018** | Boy | 43 | 33 | Significant difference (P<0.05). |
| | Girl | 71 | 101 | |
| | No idea | 343 | 257 | |

*A18-Age at the first child birthed*

| Author | Group | SFI | Non-SFI | Results |
|---|---|---|---|---|
| **Yuan,2020** | <=30 | 192 | 259 | Significant difference ($\chi^2$=15.427, P<0.01). |
| | >30 | 30 | 84 | |

*A19- Expected sex of first baby*

| Author | Group | SFI | Non-SFI | Results |
|---|---|---|---|---|
| **Hua,2021** | Boy | 32 | 22 | No significant difference ($\chi^2$ test). |
| | Girl | 44 | 30 | |
| | No idea | 39 | 15 | |

*A20- Expected sex of two babies*

| Author | Group | SFI | Non-SFI | Results |
|---|---|---|---|---|
| **Hua,2021** | Two girls | 23 | 17 | No significant difference ($\chi^2$ test). |
| | Two boys | 43 | 19 | |
| | Boy and Girl | 39 | 31 | |

*A21- The impact of fertility on women's career development*

| Author | Group | SFI | Non-SFI | Results |
|---|---|---|---|---|
| **Hua,2021** | No effect | 29 | 19 | No significant difference ($\chi^2$ test). |
| | Influence career choices | 43 | 24 | |
| | Influence promotion and salary increase | 33 | 24 | |

| | | | | |
|---|---|---|---|---|
| **Fan,2021** | Very high | 9 | 47 | Significant difference ($\chi^2$=24.472, P<0.001). |
| | High | 29 | 40 | |
| | Not too bad | 53 | 123 | |
| | Low | 77 | 83 | |
| | Very low | 21 | 26 | |

***A22-Facing conflicting choices between career and fertility***

| Author | Group | SFI | Non-SFI | Results |
|---|---|---|---|---|
| **Hua,2021** | Choose children | 50 | 30 | Significant difference ($\chi^2$=37.718, P<0.001). |
| | Choose career | 30 | 15 | |
| | Balancing children and career | 25 | 22 | |
| **Ji,2019** | Choose children | 22 | 21 | Significant difference ($\chi^2$=27.392, P<0.001). |
| | Choose career | 12 | 81 | |
| | Balancing children and career | 56 | 97 | |
| | Others | 1 | 10 | |

***B1- Husband's age***

| Author | Group | SFI | Non-SFI | Results |
|---|---|---|---|---|
| **Qin,2018** | 20-29 | 20 | 32 | No significant difference ($\chi^2$ test). |
| | 30-39 | 29 | 102 | |
| | 40-49 | 7 | 21 | |
| **Wang T,2019** | <31 | 46 | 16 | Significant difference ($\chi^2$=74.66, P<0.001). |
| | 31-35 | 54 | 23 | |
| | >35 | 128 | 263 | |

***B2- Husband's education***

| Author | Group | SFI | Non-SFI | Results |
|---|---|---|---|---|
| **Hui,2019** | Less than primary school | 1 | 24 | Significant difference ($\chi^2$=35.842, P<0.001). |
| | Junior high school | 28 | 461 | |
| | High school or technical secondary school | 66 | 1400 | |
| | Junior college | 164 | 2828 | |
| | Bachelor degree | 259 | 4177 | |
| | Master and above | 116 | 1090 | |
| **Qin,2018** | Junior high school and below | 10 | 30 | No significant difference ($\chi^2$ test). |
| | High school or college degree | 19 | 79 | |
| | Bachelor and above | 27 | 48 | |
| **Bai,2018** | High school and below | 74 | 82 | Significant difference (P<0.01). |
| | College degree or bachelor degree | 258 | 236 | |
| | Master and above | 85 | 42 | |

| | | | | |
|---|---|---|---|---|
| **Wang T,2019** | Less than high school | 30 | 73 | Significant difference ($\chi^2$=13.27 , P<0.01). |
| | Junior college or college degree | 187 | 215 | |
| | Hight than bachelor's degree | 11 | 14 | |
| **Jin,2018** | Junior high school and below | 202 | 550 | No |
| | High school | 322 | 613 | |
| | College degree | 225 | 363 | |
| | Bachelor and above | 128 | 167 | |

### *B3- Husband's hukou*

| Author | Group | SFI | Non-SFI | Results |
|---|---|---|---|---|
| **Li,2017** | Rural | 55 | 63 | Significant difference ($\chi^2$=4.613, P<0.05). |
| | Non-rural | 115 | 210 | |

### *B4- The husband is a local*

| Author | Group | SFI | Non-SFI | Results |
|---|---|---|---|---|
| **Hui,2019** | Yes | 393 | 7467 | Significant difference ($\chi^2$=53.197, P<0.001). |
| | No | 246 | 2544 | |
| **Li,2017** | Yes | 102 | 200 | Significant difference ($\chi^2$=8.489, P<0.01). |
| | No | 68 | 73 | |
| **Qin,2018** | Yes | 43 | 126 | No significant difference ($\chi^2$ test). |
| | No | 13 | 31 | |

### *B5- Husband's occupation*

| Author | Group | SFI | Non-SFI | Results |
|---|---|---|---|---|
| **Hui,2019** | Heads of government department or public institution | 55 | 706 | No significant difference ($\chi^2$ test). |
| | Professional skill worker | 225 | 3521 | |
| | Civil servant or clerk | 65 | 1090 | |
| | business or service workers | 137 | 2421 | |
| | Agriculture, forestry, animal husbandry and fishery workers | 2 | 25 | |
| | Production transport operator | 39 | 704 | |
| | Freelancers | 71 | 950 | |
| | Others | 34 | 439 | |
| **Qin,2018** | Employed | 36 | 95 | No significant difference ($\chi^2$ test). |
| | Self employed | 18 | 53 | |
| | Unemployed | 2 | 9 | |

### *B6- Husband's job stability*

| Author | Group | SFI | Non-SFI | Results |
|---|---|---|---|---|

| | | | | |
|---|---|---|---|---|
| **Hui,2019** | Government Department or Public Institution | 85 | 1111 | No significant difference ($\chi^2$ test). |
| | State-owned enterprise or Collective enterprises | 124 | 2069 | |
| | Private enterprise | 221 | 3500 | |
| | Foreign company or joint venture | 114 | 1989 | |
| | Individually-owned business | 58 | 660 | |
| | Army | 4 | 39 | |
| | Others | 12 | 201 | |
| | Freelancers | 12 | 246 | |

### *B7- Husband's income*

| Author | Group | SFI | Non-SFI | Results |
|---|---|---|---|---|
| **Qin,2018** | <5000 | 11 | 64 | Significant difference ($\chi^2$=12.641, P<0.01). |
| | 5000-6999 | 16 | 50 | |
| | >=7000 | 29 | 43 | |

### *B8- Whether husband from only one child family*

| Author | Group | SFI | Non-SFI | Results |
|---|---|---|---|---|
| **Qin,2018** | Yes | 16 | 49 | No significant difference ($\chi^2$ test). |
| | No | 40 | 108 | |
| **Jin,2018** | Yes | 254 | 380 | No |
| | No | 481 | 1102 | |

### *B9- Fertility attitude of husband*

| Author | Group | SFI | Non-SFI | Results |
|---|---|---|---|---|
| **Wang T,2019** | Support | 169 | 52 | Significant difference ($\chi^2$=175.77, P<0.001). |
| | Non-support | 15 | 114 | |
| | No idea | 40 | 104 | |

### *B10- Husband 's desired number of children*

| Author | Group | SFI | Non-SFI | Results |
|---|---|---|---|---|
| **Jin,2018** | <=1 | 105 | 889 | No |
| | >1 | 772 | 804 | |

### *B11- Household registration type of the couple*

| Author | Group | SFI | Non-SFI | Results |
|---|---|---|---|---|
| **Tao,2021** | Rural residents | 722 | 406 | Significant difference (P<0.05). |
| | One rural and one urban | 70 | 45 | |
| | Urban residents | 22 | 34 | |

### *B12- Whether the couple comes from only one-child family*

| Author | Group | SFI | Non-SFI | Results |
|---|---|---|---|---|

| | | | | |
|---|---|---|---|---|
| **Yuan,2021** | One among the couple was the only child | 285 | 130 | Significant difference ($\chi^2$=33.471, P<0.001). |
| | Both members of a couple were the only child | 71 | 86 | |
| | Others | 227 | 124 | |
| **Hua,2021** | Wife was the only child | 33 | 17 | Significant difference ($\chi^2$=31.501, P<0.001). |
| | The husband was the only child | 17 | 15 | |
| | Both members of a couple were the only child | 24 | 21 | |
| | Others | 31 | 14 | |
| **Liang,2019** | One among the couple was the only child | 50 | 137 | Significant difference ($\chi^2$=19.570, P<0.001). |
| | Both members of a couple were the only child | 111 | 233 | |
| | Others | 26 | 152 | |
| **Bai,2018** | One among the couple was the only child | 129 | 110 | Significant difference (P<0.01). |
| | Both members of a couple were the only child | 152 | 165 | |
| | Others | 136 | 85 | |
| **Wang T,2019** | One among the couple was the only child | 57 | 45 | Significant difference ($\chi^2$=20.85, P<0.001). |
| | Both members of a couple were the only child | 21 | 13 | |
| | Others | 150 | 244 | |

***B13- Annual household income***

| Author | Group | SFI | Non-SFI | Results |
|---|---|---|---|---|
| **Yuan,2020** | <50000 | 34 | 99 | Significant difference ($\chi^2$=62.474, P<0.001). |
| | 50000-100000 | 83 | 191 | |
| | 100000-150000 | 78 | 105 | |
| | 150000-200000 | 52 | 49 | |
| | >=200000 | 60 | 31 | |
| **Liang,2019** | <100000 | 29 | 81 | Significant difference ($\chi^2$=16.736, P<0.05). |
| | 100000-190000 | 62 | 226 | |
| | 200000-290000 | 49 | 125 | |
| | 300000-490000 | 32 | 68 | |
| | >=500000 | 15 | 22 | |
| **Hui,2019** | <100000 | 86 | 1437 | Significant difference ($\chi^2$=46.035, P<0.001). |
| | 100000-200000 | 161 | 3463 | |
| | 200000-300000 | 151 | 2505 | |
| | >=300000 | 225 | 2421 | |

### *B14-Family financial self-assessment*

| Author | Group | SFI | Non-SFI | Results |
|---|---|---|---|---|
| **Qin,2018** | Low | 15 | 62 | No significant difference ($\chi^2$ test). |
| | Medium | 26 | 54 | |
| | High | 15 | 41 | |
| **Zhao,2019** | Low | 270 | 360 | No significant difference ($\chi^2$ test). |
| | Medium | 264 | 362 | |
| | High | 62 | 128 | |
| **Jin,2018** | 0-25% | 211 | 426 | No |
| | 25%-50% | 167 | 494 | |
| | 50%-75% | 213 | 440 | |
| | 75%-100% | 286 | 334 | |

### *B15- House property*

| Author | Group | SFI | Non-SFI | Results |
|---|---|---|---|---|
| **Ji,2019** | Own house | 84 | 174 | No significant difference ($\chi^2$ test). |
| | Rent | 2 | 24 | |
| | Affordable housing | 1 | 0 | |
| | Company provides housing | 1 | 4 | |
| | Others | 3 | 7 | |
| **Lin,2019** | Parents' house | 90 | 35 | No significant difference ($\chi^2$ test). |
| | House owned by loan | 57 | 31 | |
| | Own house | 49 | 23 | |
| | Others | 19 | 7 | |
| **Qin,2018** | Own house | 25 | 68 | No significant difference ($\chi^2$ test). |
| | Others | 31 | 89 | |

### *B16- Live together with parents*

| Author | Group | SFI | Non-SFI | Results |
|---|---|---|---|---|
| **Jin,2018** | No | 614 | 1271 | No |
| | live with parents | 65 | 103 | |
| | live with in-laws | 195 | 319 | |
| **Zhao,2019** | No | 394 | 442 | No significant difference ($\chi^2$ test). |
| | live with parents | 89 | 249 | |
| | live with in-laws | 113 | 159 | |
| **Lin,2019** | No | 22 | 35 | Significant difference ($\chi^2$=49.675, |
| | Yes | 194 | 60 | $P<0.001$). |

### *B17- Who influences fertility intention*

| Author | Group | SFI | Non-SFI | Results |
|---|---|---|---|---|

| | | | | |
|---|---|---|---|---|
| **Hua,2021** | Husband | 22 | 9 | No significant difference ($\chi^2$ test). |
| | Self | 28 | 21 | |
| | Parents | 27 | 15 | |
| | Parents in law | 24 | 18 | |
| | Child | 4 | 4 | |

*B18- Marital satisfaction*

| Author | Group | SFI | Non-SFI | Results |
|---|---|---|---|---|
| **Lin,2019** | Satisfied | 147 | 62 | No significant difference ($\chi^2$ test). |
| | Not too bad | 61 | 31 | |
| | Dissatisfied | 7 | 3 | |

*B19- Number of children being raised*

| Author | Group | SFI | Non-SFI | Results |
|---|---|---|---|---|
| **Yuan,2021** | 0 | 254 | 24 | Significant difference ($\chi^2$=330.931, P<0.001). |
| | 1 | 212 | 315 | |
| | 2 | 117 | 1 | |
| **Bai,2018** | 0 | 184 | 127 | Significant difference (P<0.05). |
| | 1 | 273 | 264 | |

*B20-Sex of children being raised*

| Author | Group | SFI | Non-SFI | Results |
|---|---|---|---|---|
| **Yuan,2021** | Male | 150 | 160 | Significant difference ($\chi^2$=38.495, P<0.001). |
| | Male and female | 36 | 0 | |
| | Female | 141 | 156 | |
| **Huang,2019** | Male | 66 | 74 | No significant difference ($\chi^2$test). |
| | Female | 67 | 61 | |

*B21- Age of first child*

| Author | Group | SFI | Non-SFI | Results |
|---|---|---|---|---|
| **Zhao,2019** | <=2 | 404 | 458 | Significant difference ($\chi^2$=32.760, P<0.001). |
| | 2-3 | 190 | 594 | |
| | 3-7 | 130 | 186 | |
| | >7 | 466 | 664 | |
| **Hui,2019** | 0-1 | 639 | 10050 | No significant difference ($\chi^2$ test). |
| | 1-2 | 150 | 2588 | |
| | 2-3 | 262 | 3640 | |
| | 3-4 | 224 | 3770 | |
| | >=4 | 1 | 28 | |
| **Wang T,2019** | <6 | 99 | 40 | Significant difference ($\chi^2$=118.20, P<0.001). |
| | 6-12 | 85 | 87 | |
| | >12 | 44 | 175 | |

*B22- Gender of first child*

| Author | Group | SFI | Non-SFI | Results |
|---|---|---|---|---|
| **Yuan,2020** | Male | 102 | 187 | Significant difference ($\chi^2$=6.045, P<0.05). |
| | Female | 120 | 157 | |
| **Zhao,2019** | Male | 151 | 458 | Significant difference ($\chi^2$=4.42, P<0.05). |
| | Female | 194 | 229 | |
| **Lin,2019** | Male | 84 | 34 | Significant difference ($\chi^2$=29.163, P<0.001). |
| | Female | 77 | 14 | |
| **Qin,2018** | Male | 19 | 88 | Significant difference ($\chi^2$=5.441, P<0.05). |
| | Female | 30 | 64 | |
| **Tao,2021** | Male | 568 | 380 | Significant difference (P<0.05). |
| | Female | 246 | 105 | |
| **Jin,2018** | Male | 458 | 994 | No |
| | Female | 420 | 699 | |

*B23- The health condition of first child*

| Author | Group | SFI | Non-SFI | Results |
|---|---|---|---|---|
| **Lin,2019** | Good | 198 | 29 | Significant difference ($\chi^2$=31.731, P<0.001). |
| | Not too bad | 16 | 5 | |
| | Bad | 0 | 1 | |
| | Worst | 1 | 1 | |

*B24-The way of children care*

| Author | Group | SFI | Non-SFI | Results |
|---|---|---|---|---|
| **Hua,2021** | By myself | 51 | 25 | Significant difference ($\chi^2$=115.729, P<0.001). |
| | By parents | 22 | 21 | |
| | By nanny | 25 | 13 | |
| | Others | 7 | 8 | |
| **Lin,2019** | By myself | 117 | 49 | Significant difference ($\chi^2$=30.173, P<0.001). |
| | By parents | 73 | 26 | |
| | By parents-in-law | 14 | 12 | |
| | Others | 11 | 9 | |

*B25-The cost of raising children*

| Author | Group | SFI | Non-SFI | Results |
|---|---|---|---|---|
| **Hua,2021** | Highest | 45 | 33 | Significant difference ($\chi^2$=27.788, P<0.01). |
| | Higher | 37 | 21 | |
| | Not too low | 13 | 7 | |
| | Lower | 8 | 5 | |
| | Lowest | 2 | 1 | |

| | | | | |
|---|---|---|---|---|
| **Ji,2019** | Highest | 12 | 13 | Significant difference ($\chi^2$=15.941, P<0.01). |
| | Higher | 22 | 39 | |
| | Not too low | 34 | 55 | |
| | Lower | 6 | 10 | |
| | Lowest | 12 | 76 | |

*B26-Babysitting fees*

| Author | Group | SFI | Non-SFI | Results |
|---|---|---|---|---|
| **Hui,2019** | 0-1999 | 1 | 7 | No significant difference ($\chi^2$ test). |
| | 2000-3999 | 6 | 78 | |
| | 4000-5999 | 16 | 172 | |
| | 6000-7999 | 17 | 135 | |
| | >7999 | 2 | 38 | |

*B27- Childcare costs*

| Author | Group | SFI | Non-SFI | Results |
|---|---|---|---|---|
| **Hui,2019** | 0-1999 | 88 | 1536 | Significant difference ($\chi^2$=19.031, P<0.01). |
| | 2000-3999 | 139 | 1965 | |
| | 4000-5999 | 55 | 582 | |
| | 6000-7999 | 11 | 77 | |
| | 8000-9999 | 5 | 23 | |
| | >9999 | 5 | 49 | |

*B28-Husband's time spent on housework and child care*

| Author | Group | SFI | Non-SFI | Results |
|---|---|---|---|---|
| **Zhao,2019** | <=1h | 226 | 396 | Significant difference ($\chi^2$=9.01, P<0.01). |
| | >1h | 370 | 454 | |

*B29-Fertility attitudes of the first child*

| Author | Group | SFI | Non-SFI | Results |
|---|---|---|---|---|
| **Wang T,2019** | Support | 142 | 73 | Significant difference ($\chi^2$=94.43, P<0.001). |
| | Non-support | 13 | 77 | |
| | No idea | 64 | 125 | |

*B30- Parents support*

| Author | Group | SFI | Non-SFI | Results |
|---|---|---|---|---|
| **Wang T,2019** | Yes | 84 | 67 | Significant difference ($\chi^2$=20.86, P<0.001). |
| | No | 144 | 235 | |
| **Li,2017** | Yes | 142 | 200 | Significant difference ($\chi^2$=6.277, P<0.05). |
| | No | 28 | 73 | |
| **Jin,2018** | Yes | 618 | 795 | No |
| | No | 258 | 898 | |

*B31- To have second child pressure from parents*

| Author | Group | SFI | Non-SFI | Results |
|---|---|---|---|---|

| | | | | |
|---|---|---|---|---|
| **Lin,2019** | Yes | 49 | 18 | No significant difference |
| | No | 166 | 78 | ($\chi^2$ test). |

### *B32- Parent's fertility attitude*

| Author | Group | SFI | Non-SFI | Results |
|---|---|---|---|---|
| **Wang T,2019** | Support | 184 | 109 | Significant difference |
| | Non-support | 6 | 39 | ($\chi^2$=97.18, P<0.001). |
| | No idea | 29 | 122 | |

### *B33- Parents' desired number of children*

| Author | Group | SFI | Non-SFI | Results |
|---|---|---|---|---|
| **Jin,2018** | <=1 | 30 | 469 | No |
| | >=2 | 782 | 1067 | |
| | No idea | 65 | 156 | |

### *B34- Whether the first child's gender matches the parents' expectations*

| Author | Group | SFI | Non-SFI | Results |
|---|---|---|---|---|
| **Jin,2018** | Yes | 692 | 1463 | No |
| | No | 185 | 229 | |

### *B35-In-laws' fertility attitude*

| Author | Group | SFI | Non-SFI | Results |
|---|---|---|---|---|
| **Wang T,2019** | Support | 170 | 91 | Significant difference |
| | Non-support | 2 | 29 | ($\chi^2$=93.31, P<0.001). |
| | No idea | 46 | 141 | |

### *B36-In-law relationships*

| Author | Group | SFI | Non-SFI | Results |
|---|---|---|---|---|
| **Li,2017** | Best | 36 | 42 | No significant difference |
| | Good | 83 | 154 | ($\chi^2$ test). |
| | Not too bad | 48 | 64 | |
| | Bad | 3 | 11 | |
| | Worst | 0 | 2 | |

### *B37-Envy of others people's second child*

| Author | Group | SFI | Non-SFI | Results |
|---|---|---|---|---|
| **Lin,2019** | Yes | 95 | 27 | Significant difference |
| | No | 120 | 69 | ($\chi^2$=7.181, P<0.01). |

### *B38-Friend's fertility attitude*

| Author | Group | SFI | Non-SFI | Results |
|---|---|---|---|---|
| **Wang T,2019** | Support | 120 | 20 | Significant difference |
| | Non-support | 6 | 26 | ($\chi^2$=144.49, P<0.001). |
| | No idea | 81 | 216 | |

### *C1- The attitude of women's companies*

| Author | Group | SFI | Non-SFI | Results |
|---|---|---|---|---|
| **Hua,2021** | Not supported at all | 15 | 11 | Significant difference ($\chi^2$=29.789, P<0.001). |
| | Non-support | 25 | 16 | |
| | Not too supportive | 26 | 16 | |
| | Support | 31 | 19 | |
| | Fully support | 8 | 5 | |
| **Ji,2019** | Not supported at all | 2 | 13 | Significant difference ($\chi^2$=12.945, P<0.05). |
| | Non-support | 6 | 18 | |
| | Not too supportive | 39 | 127 | |
| | Support | 17 | 20 | |
| | Fully support | 27 | 31 | |

*C2- Women's awareness of the universal two-child policy*

| Author | Group | SFI | Non-SFI | Results |
|---|---|---|---|---|
| **Hua,2021** | Unknown | 11 | 3 | Significant difference ($\chi^2$=40.883, P<0.001). |
| | Not too understand | 22 | 25 | |
| | Understand | 49 | 25 | |
| | Fully understand | 23 | 14 | |
| **Ji,2019** | Unknown | 2 | 18 | No significant difference ($\chi^2$ test). |
| | Not too understand | 16 | 45 | |
| | Understand | 51 | 104 | |
| | Fully understand | 22 | 42 | |

*C3- Women's fertility intention before the implementation of the universal two-child policy*

| Author | Group | SFI | Non-SFI | Results |
|---|---|---|---|---|
| **Hua,2021** | No | 47 | 42 | Significant difference ($\chi^2$=7.464, P<0.05). |
| | Yes | 58 | 25 | |

*C4- The perfection of medical and pension insurance in public places*

| Author | Group | SFI | Non-SFI | Results |
|---|---|---|---|---|
| **Hua,2021** | Imperfect | 27 | 35 | Significant difference ($\chi^2$=26.546, P<0.001). |
| | Not too complete | 18 | 29 | |
| | Perfect | 22 | 41 | |

*C5-The state of supervision of child care institutions*

| Author | Group | SFI | Non-SFI | Results |
|---|---|---|---|---|
| **Ji,2019** | Unregulated | 12 | 39 | Significant difference ($\chi^2$=5.383, P<0.05). |
| | Regulated | 79 | 79 | |

**Table S6 Quality scores assessing risk of bias using a modified Newcastle-Ottawa scale**

| Study type: Cross-sectional; Score: 1=achieved, 0=not achieved | | | | | | | | |
|---|---|---|---|---|---|---|---|---|
| **Authors** | **Representativeness of the sample** | **Sample size** | **Non-respondents** | **Ascertainment of the exposure** | **Comparability of subjects in different outcome groups (control for confounding)** | **Assessment of the outcome** | **Statistical test is appropriate** | **Total score** |
| 213Yuan,2021 | 1 | 1 | 0 | 1 | 1 | 1 | 1 | 6 |
| 186Hua,2021 | 1 | 0 | 0 | 1 | 1 | 1 | 1 | 5 |
| 177Fan,2021 | 0 | 0 | 0 | 1 | 1 | 1 | 1 | 4 |
| 162Yang,2020 | 0 | 0 | 0 | 1 | 0 | 1 | 0 | 2 |
| 144 Yuan,2020 | 1 | 1 | 0 | 1 | 1 | 1 | 1 | 6 |
| 137Ji,2019 | 1 | 0 | 0 | 1 | 0 | 1 | 1 | 4 |
| 134Huang,2019 | 1 | 0 | 0 | 1 | 1 | 1 | 0 | 4 |
| 132Zhao,2019 | 1 | 1 | 0 | 1 | 1 | 1 | 1 | 6 |
| 128Lin,2019 | 0 | 0 | 0 | 1 | 1 | 1 | 1 | 4 |
| 121Hui,2019 | 1 | 1 | 0 | 1 | 1 | 1 | 1 | 6 |
| 109Li,2017 | 1 | 0 | 0 | 1 | 1 | 1 | 1 | 5 |
| 95Liang,2019 | 1 | 1 | 0 | 1 | 1 | 1 | 1 | 6 |
| 87Qin,2018 | 0 | 0 | 0 | 1 | 1 | 1 | 1 | 4 |
| 83Jin,2018 | 1 | 1 | 0 | 1 | 1 | 1 | 0 | 5 |
| 62Tao,2021 | 1 | 1 | 0 | 1 | 0 | 1 | 1 | 5 |
| 21Bai,2018 | 0 | 1 | 0 | 1 | 0 | 1 | 1 | 4 |
| 4Wang T,2019 | 1 | 0 | 0 | 1 | 1 | 1 | 1 | 5 |

**Table S7 Meta-analyses on 18 factors**

| Exposure | No. of studies in Meta-analyses | Comparison model | Q test | | Statistical model | Pooled OR | Z test | Egger |
|---|---|---|---|---|---|---|---|---|
| | | | I² | *P*-value | | OR (95%CI) | *P*-value | P>\|t\| |
| **Individual level** | | | | | | | | |
| **Demographic factors** | | | | | | | | |
| Age | 9 | Younger(<=35 years old) vs. Older(>35 years old) | 89% | <0.00001 | Random-effect | 2.90 [2.12,3.96] | <0.00001 | 0.313 |
| Education | 8 | High school and below vs. Junior college or above | 39% | 0.12 | Fixed-effect | 0.68 [0.62,0.75] | <0.00001 | 0.456 |
| Hukou | 3 | Rural vs. Urban | 85% | 0.001 | Random-effect | 1.55 [0.79,3.05] | 0.20 | 0.566 |
| Income | 3 | Low-income(<=8000 yuan) vs. High-income(>8000 yuan) | 65% | 0.06 | Random-effect | 0.68 [0.46,0.99] | 0.04 | 0.803 |
| Marital status | 3 | Unmarried vs. Married | 98% | <0.00001 | Random-effect | 1.37 [0.12,16.04] | 0.80 | 0.231 |
| Work stability | 4 | High vs. Low | 0% | 0.41 | Fixed-effect | 1.33 [1.06,1.66] | 0.01 | 0.528 |
| **Fertility attitude** | | | | | | | | |
| Ideal number of children | 3 | <=1 vs. >1 | 91% | <0.00001 | Random-effect | 0.04 [0.01,0.18] | <0.0001 | 0.171 |
| Gender preference | 3 | Yes vs. No | 92% | <0.00001 | Random-effect | 1.20 [0.51,2.81] | 0.67 | 0.502 |
| **Family level** | | | | | | | | |
| **Husband factors** | | | | | | | | |

| | | | | | | | | |
|---|---|---|---|---|---|---|---|---|
| Husband's education | 5 | High school and below vs. Junior college or above | 17% | 0.30 | Fixed-effect | 0.68 [0.60,0.77] | <0.00001 | 0.210 |
| The husband has a local hukou | 3 | Yes vs. No | 0% | 0.58 | Fixed-effect | 0.55 [0.48,0.64] | <0.00001 | 0.403 |
| Whether the couple comes from only one-child family | 3 | Yes vs. No | 30% | 0.24 | Fixed-effect | 0.76 [0.62,0.93] | 0.008 | 0.683 |
| Annual household income | 3 | Low(<200000 yuan) vs. High(>=200000 yuan) | 82% | 0.003 | Random-effect | 0.52 [0.34,0.80] | 0.003 | 0.450 |
| Family financial self-assessment | 3 | Low-level vs. High-level | 90% | <0.00001 | Random-effect | 0.89 [0.54,1.46] | 0.63 | 0.744 |
| House property | 3 | Yes vs. No | 44% | 0.17 | Fixed-effect | 1.27 [0.81,1.96] | 0.29 | 0.846 |
| Live together with parents | 3 | Yes vs. No | 97% | <0.00001 | Random-effect | 0.69 [0.29,1.63] | 0.39 | 0.632 |
| **Children factors** | | | | | | | | |
| Age of first child | 3 | Before kindergarten(<=3 years old) vs. After kindergarten(>3 years old) | 93% | <0.00001 | Random-effect | 1.32 [0.83,2.10] | 0.25 | 0.334 |
| Gender of first child | 6 | Male vs. Female | 76% | 0.001 | Random-effect | 0.58 [0.44,0.75] | <0.0001 | 0.310 |
| **Parents or others factors** | | | | | | | | |
| Parents support | 4 | Yes vs. No | 79% | 0.002 | Random-effect | 1.88 [1.26,2.80] | 0.002 | 0.113 |

**Table S8 Factors not included in the meta-analysis and whether they were significantly associated with SFI**

| Group | Category | Factors | Authors | Result |
|---|---|---|---|---|
| **Individual level** | **Demographic factors** | A7- Hold a management position | Hua,2021 | No significant difference ($\chi^2$test). |
| | | A8-Job title | Yuan,2020 | No significant difference ($\chi^2$test). |
| | | A9- Nationality | Huang,2019 | No significant difference ($\chi^2$test). |
| | | A10-Whether comes from only one child family | Jin,2018 | No |
| | | | Lin,2019 | Significant difference ($\chi^2$=9.158, P<0.01). |
| | | A11- Physical health | Fan,2021 | Significant difference ($\chi^2$=10.851, P<0.05). |
| | | | Lin,2019 | No significant difference ($\chi^2$ test). |
| | | A12- Frequency of participation in community activities | Zhao,2019 | Significant difference ($\chi^2$=22.78, P<0.001). |
| | | A13- Employed | Jin,2018 | No |
| | | A14- Work pressure | Li,2017 | Significant difference ($\chi^2$=-3.177[a], P<0.01). |
| | **Fertility attitude** | A15- Ideal childbearing age | Lin,2019 | Significant difference ($\chi^2$=12.584, P<0.01) |

| | | | | |
|---|---|---|---|---|
| | | A18- Age at the first child birthed | Yuan,2020 | Significant difference ($\chi^2$=15.427, P<0.01). |
| | | A19- Expected sex of first child | Hua,2021 | No significant difference ($\chi^2$ test). |
| | | A20- Expected sex of two children | Hua,2021 | No significant difference ($\chi^2$ test). |
| | | A21- The impact of childbirth on women's careers | Hua,2021<br>Fan,2021 | No significant difference ($\chi^2$ test).<br>Significant difference ($\chi^2$=24.472, P<0.001). |
| | | A22- Facing conflicting choices between career and fertility | Hua,2021<br>Ji,2019 | Significant difference ($\chi^2$=37.718, P<0.001).<br>Significant difference ($\chi^2$=27.392, P<0.001). |
| **Family level** | **Husband factors** | B1-Husband's age | Qin,2018<br>Wang T,2019 | No significant difference ($\chi^2$ test).<br>Significant difference ($\chi^2$=74.66, P<0.001). |
| | | B3- Husband's hukou | Li,2017 | Significant difference ($\chi^2$=4.613, P<0.05). |
| | | B5- Husband's occupation | Hui,2019<br>Qin,2018 | No significant difference ($\chi^2$ test).<br>No significant difference ($\chi^2$ test). |
| | | B6- Husband's job stability | Hui,2019 | No significant difference ($\chi^2$ test). |

| | | | |
|---|---|---|---|
| | B7- Husband's income | Qin,2018 | Significant difference ($\chi^2$=12.641, P<0.01). |
| | B8-Whether husband from only one child family | Qin,2018 | No significant difference ($\chi^2$ test). |
| | | Jin,2018 | No |
| | B9- Fertility attitude of husband | Wang T,2019 | Significant difference ($\chi^2$=175.77, P<0.001). |
| | B10- Husband's desired number of children | Jin,2018 | No |
| | B11- Household registration type of the couple | Tao,2021 | Significant difference (P<0.05). |
| | B17- Who influences fertility intention | Hua,2021 | No significant difference ($\chi^2$ test). |
| | B18-Marital satisfaction | Lin,2019 | No significant difference ($\chi^2$ test). |
| **Children factors** | B19-Number of children being raised | Yuan,2020 | Significant difference ($\chi^2$=330.931, P<0.001). |
| | | Bai,2018 | Significant difference (P<0.05). |
| | B20- Sex of children being raised | Yuan,2021 | Significant difference ($\chi^2$=38.495, P<0.001). |
| | | Huang,2019 | No significant difference ($\chi^2$test). |
| | B23-The health condition of first child | Lin,2019 | Significant difference ($\chi^2$=31.731, P<0.001). |

| | | | |
|---|---|---|---|
| | B24-The way of children care | Hua,2021 | Significant difference ($\chi^2$=115.729, P<0.001). |
| | | Lin,2019 | Significant difference ($\chi^2$=30.173, P<0.001). |
| | B25-The cost of raising children | Hua,2021 | Significant difference ($\chi^2$=27.788, P<0.01). |
| | | Ji,2019 | Significant difference ($\chi^2$=15.941, P<0.01). |
| | B26- Babysitting fees | Hui,2019 | No significant difference ($\chi^2$ test). |
| | B27-Childcare costs | Hui,2019 | Significant difference ($\chi^2$=19.031, P<0.01). |
| | B28-Husband's time spent on housework and child care | Zhao,2019 | Significant difference ($\chi^2$=9.01, P<0.01). |
| | B29-Fertility attitudes of the first child | Wang T,2019 | Significant difference ($\chi^2$=94.43, P<0.001). |
| **Parents or others factors** | B31-To have second child pressure from parents | Lin,2019 | No significant difference ($\chi^2$ test). |
| | B32-Parent's fertility attitude | Wang T,2019 | Significant difference ($\chi^2$=97.18, P<0.001). |
| | B33-Parents' desired number of children | Jin,2018 | No |
| | B34-Whether the first child's gender matches the parents' expectations | Jin,2018 | No |

| | | | | |
|---|---|---|---|---|
| | | B35-In-laws' fertility attitude | Wang T,2019 | Significant difference ($\chi^2$=93.31, P<0.001). |
| | | B36- In-law relationships | Li,2017 | No significant difference ($\chi^2$ test). |
| | | B37-Envy of other people's second child | Lin,2019 | Significant difference ($\chi^2$=7.181, P<0.01). |
| | | B38-Friend's fertility attitude | Wang T,2019 | Significant difference ($\chi^2$=144.49, P<0.001). |
| **Social level** | **Policy factors** | C1-The attitude of women's companies | Hua,2021 | Significant difference ($\chi^2$=29.789, P<0.001). |
| | | | Ji,2019 | Significant difference ($\chi^2$=12.945, P<0.05). |
| | | C2-Women's awareness of the universal two-child policy | Hua,2021 | Significant difference ($\chi^2$=40.883, P<0.001). |
| | | | Ji,2019 | No significant difference ($\chi^2$ test). |
| | | C3-Women's fertility intention before the implementation of the universal two-child policy | Hua,2021 | Significant difference ($\chi^2$=7.464, P<0.05). |
| | **Public service** | C4- The perfection of medical and pension insurance in public places | Hua,2021 | Significant difference ($\chi^2$=26.546, P<0.001). |
| | | C5- The state of supervision of child care institutions | Ji,2019 | Significant difference ($\chi^2$=5.383, P<0.05). |
